# Comparative Analysis of Phenotypic and Genotypic Antibiotic Susceptibility of *Pasteurella multocida* Isolated from Various Host Species in France and Hungary

**DOI:** 10.3390/antibiotics14090906

**Published:** 2025-09-08

**Authors:** Krisztina Pintér, Marianna Domán, Enikő Wehmann, Hubert Gantelet, Tibor Magyar

**Affiliations:** 1 HUN-REN Veterinary Medical Research Institute, 1143 Budapest, Hungary; pinter.krisztina@vmri.hun-ren.hu (K.P.); doman.marianna@vmri.hun-ren.hu (M.D.); wehmann.eniko@vmri.hun-ren.hu (E.W.); 2 National Laboratory of Infectious Animal Diseases, Antimicrobial Resistance, Veterinary Public Health and Food Chain Safety, University of Veterinary Medicine, 1078 Budapest, Hungary; 3 Ceva Biovac, 49070 Beaucouzé, France; hubert.gantelet@ceva.com

**Keywords:** antimicrobial resistance, disk diffusion, MIC, MDR, *Pasteurella multocida*, resistance genes, whole genome sequencing

## Abstract

**Background/Objectives**: *Pasteurella multocida* is responsible for a wide variety of animal diseases worldwide, causing major economic losses. These infections are usually treated with antibiotics; however, the emergence of multidrug-resistant (MDR) strains is increasingly hindering. Understanding antibiotic resistance in *P. multocida* is important for effective treatment strategies and public health, as it impacts both animal and human welfare. **Methods**: In this study, the antibiotic susceptibility of 80 *P. multocida* isolates was evaluated by phenotypic (disk diffusion and broth microdilution) and genotypic analysis via whole-genome sequencing, with particular attention to the occurrence of MDR strains. The strains were tested against antibiotics from nine antimicrobial classes (penicillins, cephalosporins, aminoglycosides, tetracyclines, macrolides, fluoroquinolones, lincosamides, phenicols, and sulfonamides). Antimicrobial resistance gene (ARG) sequences and single-nucleotide polymorphisms (SNPs) were evaluated in paired reads using the Bacterial and Viral Bioinformatics Resource Center (BV-BRC) and the Comprehensive Antibiotic Resistance Database (CARD) via Resistance Gene Identifier (RGI), respectively. **Results**: Phenotypic results indicated that cephalosporins and phenicols were the most effective drugs against *P. multocida*; however, the majority of strains were also susceptible to fluoroquinolones and tetracyclines. In contrast, high resistance rates were observed to sulfamethoxazole and clindamycin. The most prevalent resistance genes were *str*A, *sul*2, and *tet*H, while none of the strains harbored the *bla*-_TEM_ or *erm* (42) genes. **Conclusions**: Of the two phenotypic methods, MIC values showed a stronger positive correlation with genotypic results, making it a more suitable method for determining antibiotic susceptibility. The phenotypic results for phenicols, tetracyclines, and fluoroquinolones showed a strong correlation with the detected resistance genes. In contrast, resistance to sulfamethoxazole, β-lactams, and macrolides remained genetically unexplained, suggesting the existence of additional resistance mechanisms to be explored.

## 1. Introduction

*P. multocida* is a significant veterinary pathogen with zoonotic potential, posing a risk to humans through animal contact. It is the etiological agent of fowl cholera [1], atrophic rhinitis in pigs [2], hemorrhagic septicemia in cattle and buffaloes [3], and snuffles in rabbits [4]. As an opportunistic pathogen, it is a major contributor to the development of the respiratory disease complex in cattle and swine [4]. Humans might be infected through scratches and bites from cats and dogs [5].

The spread of antimicrobial resistance is one of the most threatening global challenges of our time, and has now become one of the top 10 global health risks [6]. It is currently estimated that nearly 700,000 people die every year due to antimicrobial resistance. This could rise to 10 million by 2050, according to predictions [7]. The majority of antibiotics used to treat animal diseases are also important in human medicine. Antibiotics are essential in combating *P. multocida* infections, but the rise of multidrug-resistant (MDR) strains underscores the urgency of antimicrobial stewardship under the One Health framework [8]. The *P. multocida* strain is classified as MDR if it is resistant to at least one agent representing three or more antibiotic classes [9]. It is essential to establish a susceptibility profile before therapy in order to achieve successful treatment. There are various methods to determine the antibiotic susceptibility of strains and to detect MDR strains. Phenotypic susceptibility testing, including disk diffusion and broth microdilution method, remains the gold standard for therapy [10]. Genotypic antibiotic susceptibility testing mainly involves the identification of resistance genes (ARGs) using different polymerase chain reaction (PCR)-based methods [11]. In recent years, whole-genome sequencing has become increasingly prevalent, enabling a more efficient identification of ARGs and point mutations [12,13].

Considering the results of previous publications, the main hypotheses of this research were the following: (a) a correlation can be observed between phenotypic and genotypic resistance for each antibiotic class, (b) the broth dilution microdilution method is more reliable for phenotypic susceptibility testing than the disc diffusion method, and (c) most *P. multocida* strains are susceptible to cephalosporins, fluoroquinolones, and phenicols but resistant to sulfonamides and clindamycin. To verify the aforementioned hypotheses, the aim of this study was to determine the phenotypic and genotypic antibiotic susceptibility profiles of *P. multocida* strains, to compare the efficacy of these methods, and identify the most effective drugs for treatment.

## 2. Results

**Figure 1:**
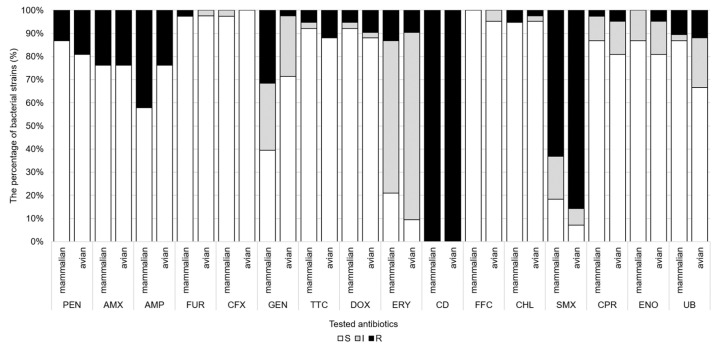
Antibiotic susceptibility rates of *P. multocida* isolates from avian and mammalian hosts, determined by disk diffusion (16 antibiotics). Abbreviations: PEN: penicillin; AMX: amoxicillin; AMP: ampicillin; FUR: ceftiofur; CFX: cephalexin; GEN: gentamicin; TTC: tetracycline; DOX: doxycycline; ERY: erythromycin; CD: clindamycin; FFC: florfenicol; CHL: chloramphenicol; SMX: sulfamethoxazole; CPR: ciprofloxacin; ENO: enrofloxacin; UB: flumequine; S: susceptible; I: intermediate; R: resistant.

The antibiotic susceptibility profiles of the tested strains obtained by disk diffusion are summarized in Figure 1. All strains were resistant to clindamycin, and with a high resistance rate (75.0%) to sulfamethoxazole. The strains were highly susceptible (>90%) to cephalosporins, tetracyclines, and phenicols. On the other hand, there was a decrease in the susceptibility of strains to penicillins (sensitivity (S): 67.5–83.8%), fluoroquinolones (S: 76.3–83.5%), and gentamicin (56.8%). A high percentage of strains (73.8%) showed intermediate susceptibility to erythromycin.

**Figure 2:**
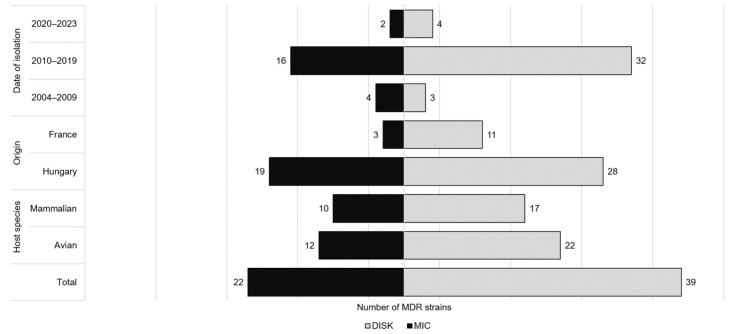
Distribution of MDR *P. multocida* isolates by host species, geographic origin, and year of isolation, based on disk diffusion and broth microdilution results.

A total of 39 MDR strains were identified, most of which showed resistance to three (*n* = 18), four (*n* = 15), and in some cases to five, six, seven, and eight antibiotic classes (Table 1). Among MDR strains, the most frequent resistance combinations were penicillins–lincosamides–sulfonamides (*n* = 8) and penicillins–aminoglycosides–lincosamides–sulfonamides (*n* = 6). Overall, these strains were most resistant to clindamycin (100%), sulfamethoxazole (95%), and ampicillin (59%). MDR strains were isolated from both avian (*n* = 22) and mammalian (*n* = 17) hosts (Figure 2).

**Figure 3:**
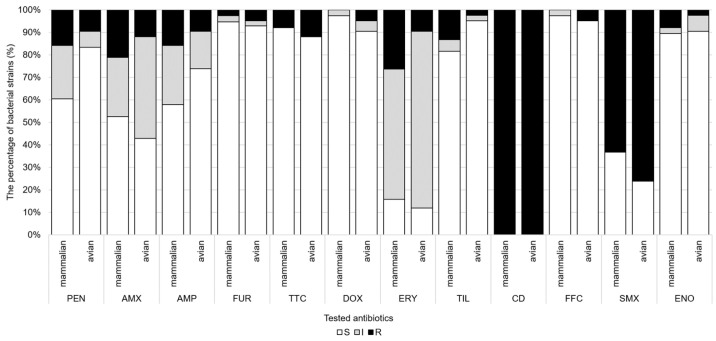
Antibiotic susceptibility rates of *P. multocida* isolates from avian and mammalian hosts, determined by broth microdilution (12 antibiotics). Abbreviations: PEN: penicillin; AMX: amoxicillin; AMP: ampicillin; FUR: ceftiofur; TTC: tetracycline; DOX: doxycycline; ERY: erythromycin; TIL: tilmicosin; CD: clindamycin; FFC: florfenicol; SMX: sulfamethoxazole; ENO: enrofloxacin; S: susceptible; I: intermediate; R: resistant.

The susceptibility data based on Minimal Inhibitory Concentration (MIC) values obtained by the broth microdilution method are summarized in Figure 3. The strains showed high susceptibility (>90%) to ceftiofur, tetracyclines, florfenicol and enrofloxacin. For these antibiotics, the calculated MIC_50_ and MIC_90_ values (Table 2) were both in the susceptible range. There was reduced susceptibility of strains to antibiotics of the penicillin class (S: 47.5–72.5%) and to tilmicosin (88.8%). For antibiotics of the penicillin class, the MIC_90_ values were always in the more resistant category. Complete resistance to clindamycin and high resistance rate (70.0%) to sulfamethoxazole were observed. The majority of strains (68.8%) had moderate susceptibility to erythromycin. The epidemiological breakpoint (ECOFF) defined by the European Committee on Antimicrobial Susceptibility Testing (EUCAST) that distinguishes wild-type and non-wild-type strains is presented in the last column of Table 2. These values were always higher than the calculated MIC_50_ values but lower than the MIC_90_ values, except for tetracyclines, tilmicosin, and florfenicol. The prevalence of non-wild-type strains was less than 10% for tetracycline, doxycycline, erythromycin, tilmicosin, and florfenicol, but higher than 50% for amoxicillin and ceftiofur. For ampicillin, enrofloxacin, and flumequine, this prevalence ranged from 17% to 33%. The susceptibility profiles of strains isolated from avian and mammalian host species are illustrated in Figure 3. A total of 22 MDR strains were identified which showed resistance to 3 (*n* = 10), 4 (*n* = 6), and 5 (*n* = 6) antibiotic classes. The most frequent resistance combination was penicillins-lincosamides-sulfonamides (*n* = 4) (Table 3). Overall, these strains were mostly resistant to clindamycin (100%), sulfamethoxazole (100%) and erythromycin (54.5%). Nearly equal proportions of MDR strains were detected from avian (54.5%) and mammalian (45.5%) hosts (Figure 2).

Whole-genome sequencing of 80 *P. multocida* isolates resulted in 1,844,984–36,016,201 reads, with average genome coverage ranging from 119× to 2295×. Metadata of the sequenced isolates, including host species and geographic origin, are detailed in Supplementary Table S1. The detection frequencies of the ARGs tested is summarized in Supplementary Table S2. None of the strains harbored the *bla*-_TEM_ or *erm* (42) resistance genes. The most prevalent resistance genes were *str*A (*n* = 7), *sul*2, and *tet*H (*n* = 6). A total of 19 strains had amino acid modifications in the *gyr*A gene. The most frequently detected amino acid substitution was Ser83Ile, affecting six strains. A total of seven strains had Ser83Arg change (five strains with AGC → AGA, and two strains with AGC → AGG single-nucleotide polymorphism (SNP)), five strains contained Asp87Asn, and one had Asp87Gly substitution. The *gyr*B gene of the tested strains did not have relevant point mutations. In the *par*C gene sequence, substitution at Glu84Lys and three strains contained an amino acid change at Ser80Leu. The most extensively resistant strain was a Hungarian bovine isolate (ID: 4221), which harbored 11 resistance genes (*te*tH, *sul*2, *msr*E, *mph*E, *str*A, *aph*A1, *str*B, *bla*-_ROB-1_, *bla*-_OXA-2_, *aad*B, and *aad*A25). However, no amino acid changes in the sequence of *gyr*A, *gyr*B, and *par*C genes responsible for fluoroquinolone resistance were identified in this strain.

A mutation within 16S rRNA conferring resistance to spectinomycin was detected in all isolates using Bacterial and Viral Bioinformatics Resource Center (BV-BRC). An SNP in 23S rRNA conferring resistance to clindamycin was found only in three isolates where the query and template sequence pairwise identity was ≥90%. No SNP was found in four strains. In the rest of the isolates, the presence of this SNP was also observed, but the pairwise sequence identity between the query sequence and the reference sequence was less than 90% (query coverage: 74.04–89.26%; reference coverage: 84.0–90.76%).

**Figure 4:**
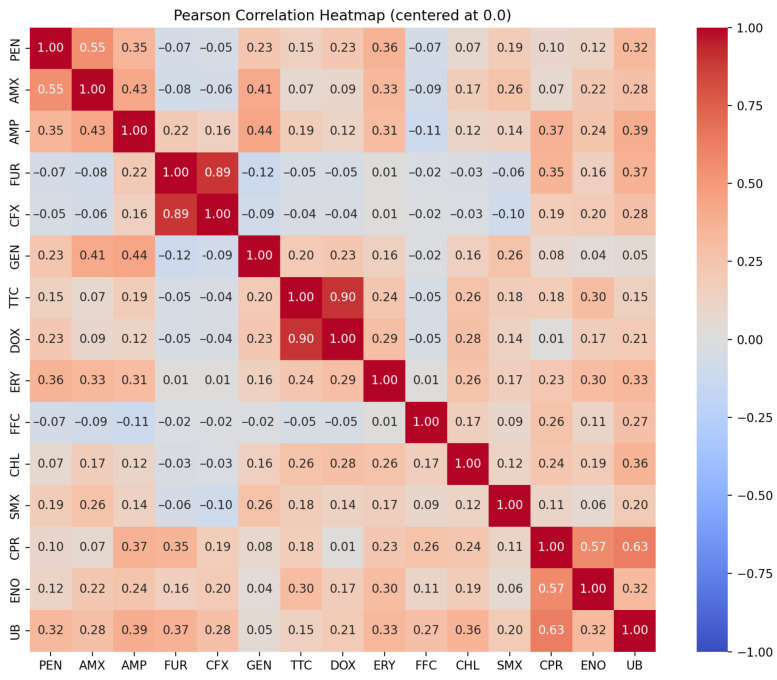
Pearson correlation heatmap based on disk diffusion results for *P. multocida* isolates. The phi values (r) range from −1 to +1, where +1 indicates a perfect positive correlation (resistance to both drugs tends to co-occur), 0 indicates no linear relationship, and −1 indicates a perfect negative correlation (resistance to one drug is associated with susceptibility to the other). Correlations were calculated using resistance data (1 = resistant; 0.5 = intermediate; 0 = susceptible) based on Clinical and Laboratory Standards Institute (CLSI) breakpoints.

According to the correlation analyses, disk diffusion results showed a strong positive correlation between antibiotics belonging to the same classes, such as tetracyclines (r = 0.9), cephalosporins (r = 0.89), and a positive correlation in the case of fluoroquinolones (r = 0.32–0.63) and penicillins (r = 0.35–0.55). In addition, correlations were observed between antibiotics belonging to the class of penicillins and gentamicin (r = 0.23–0.44), erythromycin, and flumequine (r = 0.3) (Figure 4).

**Figure 5:**
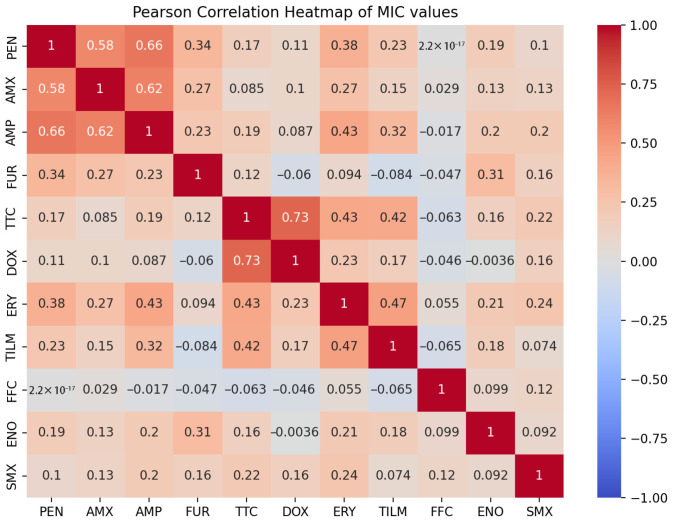
Pearson correlation heatmap based on MIC values from broth microdilution assays for *P. multocida* isolates. The phi values (r) range from −1 to +1, where +1 indicates a perfect positive correlation (resistance to both drugs tends to co-occur), 0 indicates no linear relationship, and −1 indicates a perfect negative correlation (resistance to one drug is associated with susceptibility to the other). Correlations were calculated using resistance data (1 = resistant; 0.5 = intermediate; 0 = susceptible) based on CLSI breakpoints.

The same correlations were also detected when analyzing MIC values. In addition, a correlation was noted within the class of macrolides (r = 0.47), as well as between macrolides and tetracycline (r = 0.43) (Figure 5).

**Figure 6:**
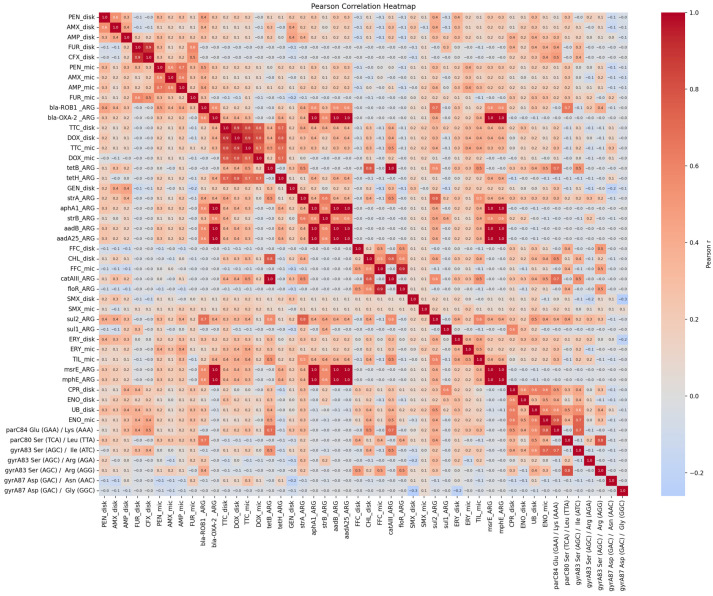
Integrated correlation heatmap showing relationships among phenotypic susceptibility, MIC values, and resistance gene/SNP presence. The phi values (r) range from −1 to +1, where +1 indicates a perfect positive correlation, 0 indicates no linear relationship, and −1 indicates a perfect negative correlation (resistance to one drug is associated with susceptibility to the other). Correlations were calculated using resistance data (1 = resistant or contain ARG/SNP; 0.5 = intermediate; 0 = susceptible) based on CLSI breakpoints.

For fluoroquinolones, a correlation was observed between phenotypically confirmed resistance and the SNPs in *par*C (Glu84Lys, r = 0.41–0.77) and in *gyr*A (Ser83Ile, r = 0.36–0.74). The strongest correlations were found between enrofloxacin MIC values and the two aforementioned SNPs (r = 0.74–0.77). The other SNPs showed no significant correlation with the phenotypic results. A negligible correlation was observed between phenotypic resistance to gentamicin and the resistance genes, whereas a strong correlation was noted between the occurrence of ARGs responsible for aminoglycoside resistance (r = 0.36–1.0). A very low correlation was detected between the phenotypic results of sulfamethoxazole, penicillins, cephalosporins, macrolides, and the ARGs responsible for resistance. In the case of tetracyclines, strong positive correlations were observed between the results of phenotypic methods and the *tet*H (r = 0.7–0.8) and a positive correlation in the case of *tet*B (r = 0.2–0.5). Similarly, the phenotypic results for florfenicol were highly correlated with the *flo*R gene (r = 0.5–0.9), and the disk diffusion results for chloramphenicol with the *cat*AIII gene (r = 0.8) (Figure 6).

## 3. Discussion

In this study, the antibiotic susceptibility profiles of 80 *P. multocida* strains were determined using phenotypic and genotypic methods. To date, phenotypic tests, primarily the disk diffusion assay, have been used to establish diagnostic and therapeutic protocols [10]. However, several studies have reported that disk diffusion is less reliable due to its low reproducibility and sensitivity, and MIC determination is preferred [14,15]. Comparing the results of the two phenotypic methods, the only significant difference was for antibiotics belonging to the penicillin class. This may be because the disk diffusion breakpoints used in this study were calculated values [16], which mainly referred to *P. multocida* strains of porcine origin, and the lack of an intermediate category made the comparison with the MIC values very limited. Between the two phenotypic tests, MIC values exhibited stronger positive correlations with genotypic findings for macrolides, enrofloxacin, and phenicols. So, in these cases, the working hypothesis was confirmed. In contrast, disk diffusion values showed higher correlation coefficients for tetracycline-class antibiotics. Both phenotypic assays showed that the majority of MDR strains were isolated during the 2010s, mainly from avian hosts in Hungary.

Disk diffusion results showed that avian strains were more susceptible to cephalosporins, whereas mammalian strains were more susceptible to fluoroquinolones, sulfamethoxazole, tetracyclines, and erythromycin. In terms of MIC values, the Hungarian strains were more susceptible to sulfamethoxazole, amoxicillin, and tetracyclines than the French isolates. Avian strains showed lower MIC values for enrofloxacin, tilmicosin, ampicillin, and penicillin than mammalian strains. These differences are probably due to different treatment protocols and antibiotics used in each country and species.

Based on the phenotypic methods, the majority of the strains were highly susceptible to phenicols. Although no resistance was detected by disk diffusion, the broth microdilution method indicated two resistant strains. These strains were of avian origin and harbored the *flo*R resistance gene, confirming the results obtained from the MIC values. Resistance to chloramphenicol was only tested by disk diffusion, which identified three resistant strains (two mammalian, one avian). Two of the strains carried the *cat*AIII gene responsible for chloramphenicol resistance, while one strain carried the *flo*R gene. The phenotypic results of florfenicol highly correlated with the *flo*R gene (r = 0.5–0.9), and the disk diffusion results of chloramphenicol with the *cat*AIII gene (r = 0.8). All resistant strains originated from Hungary. The high susceptibility of *P. multocida* strains from Hungary to phenicols has been reported previously. Sellyei et al. observed high susceptibility to florfenicol and chloramphenicol in strains of avian and porcine origin [17]. More recently, Kerek et al. have shown similarly high susceptibility to florfenicol in poultry strains [15]. Similar susceptibility profiles have been observed globally [18,19,20], although recently the number of resistant strains has been increasing [21,22,23].

The tested strains were highly susceptible to cephalosporins. Phenotypic susceptibility to cephalexin was tested by disk diffusion only, and no resistant strains were observed. Only one strain showed reduced susceptibility (ID: 4149), this bovine strain was also resistant to ceftiofur. By disk diffusion, only one strain was resistant to ceftiofur, while three resistant strains were detected by MIC testing. All these strains were originated from Hungary. Our findings are consistent with the observations previously reported in the scientific literature that cephalosporin antibiotics are highly effective for the treatment of pasteurellosis [18,19,20,22,24,25].

Within the tetracycline class, tetracycline and doxycycline were tested, and both phenotypic methods showed the latter to be more effective against the strains. Resistance to tetracycline was found in almost equal proportions by the two methods, whereas for doxycycline, the degree of resistance was higher by disk diffusion. Of the resistant or intermediate strains detected by phenotypic methods, almost all harbored *tet*H or *tet*B resistance genes. A strong positive correlation was observed between the results of phenotypic methods and the *tet*H (r = 0.7–0.8), and a positive correlation with *tet*B (r = 0.2–0.5). Overall, these results indicated that the Hungarian and French strains are essentially susceptible to tetracyclines. Resistant strains were more frequently identified from avian sources. Some authors have found similarly high levels of susceptibility [26,27]; however, the number of reports of resistance to these drugs has been increasing [19,23,24,28,29].

In general, *P. multocida* strains show high susceptibility to ciprofloxacin and enrofloxacin [18,19,20,24,26,27], but the number of resistant bacterial strains is increasing year by year [23,29]. In this study, depending on the phenotypic method, 83.8–90.0% of strains were susceptible to these antibiotics. Resistance is mainly due to mutations affecting the *gyr*A, *gyr*B, and *par*C genes. Based on literature data, the most significant amino acid changes are Ser83Ile, Asp87Gly, and Asp87Asn (*gyr*A); Glu84Lys and Ser80Leu (*par*C); and Pro415Thr and Asp426Asn (*gyr*B) [30,31,32,33,34]. No significant mutations were detected in the *gyr*B gene among the sequenced isolates. However, correlations were observed between phenotypically confirmed resistance and the SNPs *par*C Glu84Lys (r = 0.41–0.77) and *gyr*A Ser83Ile (r = 0.36–0.74). The strongest correlations were detected between the MIC values of enrofloxacin and the two aforementioned SNPs (r = 0.74–0.77). No correlation was found between other observed amino acid changes and phenotypic resistance.

Eighty-eight percent of the strains tested were susceptible to tilmicosin, and this sensitivity is globally widespread [19,24,35]. Moderate susceptibility to erythromycin is quite common in *P. multocida* strains [26,29,35], which is supported by the present observations. The *erm* (42) gene responsible for erythromycin resistance was not detected, and only a small proportion of genes responsible for other macrolide resistance were found, although a positive correlation (r = 0.4) between phenotypic and genotypic resistance was observed for tilmicosin regarding *msr*E and *mph*E. In addition to resistance genes, *P. multocida* can develop macrolide resistance through efflux mechanisms, target modification and drug inactivation, all of which can occur independently of resistance genes [36].

Based on MIC values, a total of eight strains were resistant to all three tested penicillin antibiotics. These were all Hungarian strains, the majority (75%) of which were isolated from mammals. Disk diffusion showed resistance against all penicillins in nine strains. These strains, except for one isolate, were Hungarian, mostly found in mammals. Overall, compared to the MIC values (S: 47.5–72.5%), the strains showed higher susceptibility (S: 67.5–83.8%) to these drugs using the disk diffusion method. Again, this is probably because disk diffusion breakpoints were calculated values and the lack of an intermediate category made it very difficult to compare the results with the MIC values. In a number of cases, isolates were found to be resistant to a particular penicillin antibiotic, but the genetic background for this phenotypic resistance could not be confirmed. Sahay et al. observed a similar phenomenon: by disk diffusion, 46% of the strains tested showed resistance to penicillin, but none of the isolates carried either the *bla*-_OXA-2_ or *bla*-_ROB-1_ gene [37]. In this study, the *bla*-_ROB-1_ resistance gene was only present in strains that were found to be resistant to all antibiotics in the penicillins class by both methods. Furthermore, the analysis of these antibiotics shows that *P. multocida* strains had a diverse susceptibility profile. Tang et al. reported 100% resistance to amoxicillin in porcine strains in China [28]. On the other hand, Vilaró et al. reported high susceptibility (96.2%) to amoxicillin in strains form swine in Spain [24]. Timsit et al. reported 100% penicillin susceptibility using a microdilution method, while others described 95% sensitivity [25,38]. Sabsabi et al. assessed the antibiotic susceptibility in Malaysian poultry strains using disk diffusion and found 14% resistance to both penicillin and amoxicillin [23]. Schönecker et al. observed 27% resistance to penicillin, while Xiao et al. described 28% resistance to amoxicillin [20,29].

Gentamicin is recognized worldwide as an effective drug against *P. multocida* [18,23,26,27,28]. The genotypic resistance was mainly due to genes *str*A (aminoglycoside-3-phosphotransferase), *str*B (aminoglycoside-6-phosphotransferase), *aad*A1 (aminoglycoside-3-adenyltransferase), *aad*A14 (adenyltransferase), *aph*A1 [aph (3’)-Ia] (aminoglycoside-3-phosphotransferase), *aph*A3 [aph (3’)-III], and *aad*B [ant (2”)-Ia], which may also be plasmid encoded [39,40,41]. However, gentamicin, representing aminoglycosides in this study and tested only by disk diffusion, showed 16% resistance and a relatively high rate of reduced sensitivity (27%). On the other hand, the aforementioned genes were detected only in the minority of strains. Although aminoglycoside resistance in *P. multocida* is generally associated with resistance genes, other mechanisms may also contribute to reduced drug sensitivity including ribosomal mutations or modifications, biofilm formation, and variations in virulence factors and capsular serotypes [42].

Nearly 70% of the strains were resistant to sulfamethoxazole by both phenotypic methods. French and avian strains were more resistant than Hungarian and mammalian isolates. Despite the high phenotypic resistance rate, only eight strains possessed either the *sul*1 or *sul*2 gene responsible for sulfonamide resistance. The observed phenotypic resistance rate is a widely described phenomenon [18,21,25,28]. Several publications have reported the frequent occurrence of the *sul*1 and *sul*2 genes in *P. multocida* isolates [43], typically located on various plasmids [39,44]. In addition to resistance genes, *P. multocida* can also develop sulfonamide resistance through various mechanisms, including altered membrane permeability, efflux pumps, and changes in target enzyme. These mechanisms either prevent sulfonamides from reaching their target or reduce the effectiveness of the drug [45]. Although further analyses, such as transcriptome sequencing or enzyme structure studies, were not carried out, our results indicate that these mechanisms may be rather prevalent among *P. multocida* strains than the carriage of *sul*1 and *sul*2 genes.

Absolute resistance to clindamycin was observed with both phenotypic methods. Clindamycin targets the 50S ribosomal subunit, and methylation of the adenine residues in the 23S rRNA prevents antibiotic binding to its site [46]. The point mutation in the 23S rRNA gene responsible for clindamycin resistance was identified in all but four strains using BV-BRC. High rates of resistance to clindamycin in *P. multocida* strains have been reported in several previous publications [18,26,28,29]; therefore, it is not recommended for the treatment of pasteurellosis [47].

## 4. Materials and Methods

### 4.1. Bacterial Strains

A total of 80 *P. multocida* isolates were examined, the majority of which originated from Hungary (*n* = 64) and a smaller proportion from France (*n* = 16). The strains were isolated between 2004 and 2023 from various mammalian (*n* = 38) and avian (*n* = 42) species, which were distributed among the host species as follows: cattle (*n* = 25), goose (*n* = 18), turkey (*n* = 10), duck (*n* = 10), albatross (*n* = 3), sheep (*n* = 3), goat (*n* = 3), swine (*n* = 3), dog (*n* = 1), human (*n* = 1), rabbit (*n* = 1), and fallow deer (*n* = 1). Samples were cultured on Columbia agar plates (Columbia agar, LAB M, Bury, UK) containing 5% sheep blood and incubated at 37 °C for 24 h under aerobic conditions. The identity of the strains was confirmed by PCR targeting the *kmt* gene, as described by Townsend et al. (1998) [48].

### 4.2. Antibiotic Susceptibility Testing

In vitro phenotypic susceptibility of *P. multocida* isolates was evaluated by broth microdilution method and disk diffusion against nine antibiotic classes (penicillins, cephalosporins, aminoglycosides, tetracyclines, lincosamides, macrolides, phenicols, sulfonamides, and fluoroquinolones) according to the Clinical and Laboratory Standards Institute (CLSI) guidelines [49]. The selection of these antibiotic classes was based on their common use in veterinary medicine and the need to assess resistance patterns relevant to clinical practice.

For disk diffusion, 16 antimicrobial agents (Biolab Zrt., Budapest, Hungary) were tested: penicillin G (10 U), amoxicillin (10 µg), ampicillin (10 µg), ceftiofur (30 µg), cephalexin (30 µg), gentamicin (10 µg), tetracycline (30 µg), doxycycline (30 µg), clindamycin (2 µg), erythromycin (15 µg), florfenicol (30 µg), chloramphenicol (30 µg), sulfamethoxazole (300 U), enrofloxacin (5 µg), ciprofloxacin (5 µg), and flumequine (30 µg). Results were analyzed 24 h after inoculation. Quality control was ensured using *Escherichia coli* ATCC 25922 and *Staphylococcus aureus* ATCC 25923, which served as reference strains to validate the accuracy of the antibiotic susceptibility testing.

In the broth microdilution, 14 antimicrobial agents (Sigma-Aldrich, St. Louis, MO, USA) were examined: penicillin (0.015 to 16 µg/mL), amoxicillin (0.06 to 64 µg/mL), ampicillin (0.06 to 64 µg/mL), ceftiofur (0.015 to 16 µg/mL), apramycin (0.06 to 64 µg/mL), tetracycline (0.03 to 32 µg/mL), doxycycline (0.06 to 64 µg/mL), clindamycin (0.125 to 128 µg/mL), erythromycin (0.06 to 64 µg/mL), tilmicosin (0.25 to 256 µg/mL), florfenicol (0.06 to 64 µg/mL), sulfamethoxazole (0.5 to 512 µg/mL), enrofloxacin (0.015 to 16 µg/mL), and flumequine (0.06 to 64 µg/mL). For quality control, *Escherichia coli* ATCC 25922 and *Staphylococcus aureus* ATCC 29213 were used. After incubation at 37 °C for 24 h, the turbidity (quantified) of the suspensions was measured at 450 nm using a microplate reader. The MIC was defined as the lowest concentration that completely inhibited the bacterial growth. MIC_50_ (inhibiting the growth of 50% of tested isolates) and MIC_90_ (inhibiting the growth of 90% of tested isolates) values were also calculated.

The obtained values were classified into three categories (susceptible, intermediate, or resistant) according to CLSI recommendations [50,51]. For doxycycline, the range set for tetracycline was used. For antibiotics of the penicillin class, no CLSI limit was available in the case of disk diffusion. For these antibiotics, the limits calculated by Kumakawa et al. (2025) were used [16]. A strain was considered MDR if it was resistant to at least one agent representing three or more antibiotic classes [9]. MIC values were also evaluated based on EUCAST breakpoints (https://mic.eucast.org/search/, accessed on 17 August 2025).

### 4.3. Whole Genome Sequencing and Bioinformatical Analyses

A loopful of bacteria was suspended in 2 mL of Brain Heart Infusion Broth (BHI, Merck, Darmstadt, Germany) and incubated at 37 °C for 24 h under aerobic conditions. Genomic DNA was extracted using the Quick-DNA Fungal/Bacterial Miniprep Kit (Zymo Research Corporation, Irvine, CA, USA) according to the manufacturer’s instructions. The concentration of the obtained DNA was checked using a Nanodrop spectrophotometer (Thermo Fisher Scientific, Wilmington, DE, USA). Whole-genome sequencing of the strains was accomplished by SeqOmics Biotechnology Ltd. (Mórahalom, Hungary) on the Illumina MiSeq platform (San Diego, CA, USA). Sequences read quality was assessed using FastQC (v0.12.1). Reads were trimmed with BBDuk in Geneious Prime v. 2025.0.3 [52]. De novo genome assembly was conducted with SPAdes v. 4.0.0 integrated in Geneious Prime. Genome annotation was performed using Geneious Prime and the HN07 reference genome (NZ_CP007040). To identify SNPs in the *gyr*A, *gyr*B, and *par*C genes, the sequences were aligned to the corresponding loci of the HN07 reference strain. ARGs sequences or SNPs were evaluated in paired reads using BV-BRC (https://www.bv-brc.org/) and the Comprehensive Antibiotic Resistance Database (CARD) by Resistance Gene Identifier (RGI) (https://card.mcmaster.ca/analyze/rgi, accessed on 6 May 2025), with 90% identity cut-off.

### 4.4. Statistical Analyses

Correlation analyses were performed in R 4.2.1 to investigate the relationships between the resistance observed by the two phenotypic methods and the presence of ARGs and SNPs. Pearson’s correlation matrix was used to quantify the relationships. Heatmaps were generated using Julius.AI (https://julius.ai/, accessed on 27 June 2025).

## 5. Conclusions

In conclusion, phenicols and cephalosporins were the most effective drugs against *P. multocida*, though most strains are highly susceptible to fluoroquinolones and tetracyclines as well. However, the declining susceptibility rate of fluoroquinolones is a cause for concern. Enrofloxacin is a critically important antibiotic and has a key role in human medicine. The background of decreasing susceptibility is probably due to overuse or inappropriate application; therefore, it is not recommended as first-line treatment for *P. multocida* infections in poultry considering One Health perspective. Elevated resistance rates were observed for clindamycin and sulfamethoxazole, although genotypic resistance was not always confirmed for the latter. The resistances to β-lactams and macrolides observed by phenotypic testing remains genetically unexplained and further studies, such as transcriptomic or proteomic analyses, are needed to investigate differential gene expression or protein modifications associated with resistance. Of the two phenotypic methods, MIC values showed a stronger positive correlation with genotypic results, making it a more suitable method for determining antibiotic susceptibility. This method is particularly recommended for testing antibiotics of the penicillin class for *P. multocida*. The significant economic impact of *P. multocida* infections on animal husbandry underscores the importance of continuous monitoring of antimicrobial resistance to guide treatment protocols and maintain efficacy.

## Figures and Tables

**Table 1:** Antibiotic susceptibility profiles and host species of multidrug-resistant strains identified by disk diffusion. Strains in bold were also found to be multidrug-resistant by the broth microdilution method. Abbreviations: PN: penicillins; AG: aminoglycosides; TET: tetracyclines; MAC: macrolides; LA: lincosamides; SA: sulfonamides; FC: phenicols; FQ: fluoroquinolones; CEP: cephalosporins; *n*: number of bacterial strains.

Disk Diffusion				
No. of Antibiotic Classes with Resistant Strains	No. of the Resistant Strains	Antibiotic Classes	Strain ID	Host Species (*n*)
3	3	AG-LA-SA	4373, **4380**, 4389	cattle
3	1	LA-SA-FC	**4480**	goose
3	1	LA-SA-FQ	4502	goose
3	1	MAC-LA-SA	4489	turkey
3	8	PN-LA-SA	**4376,** 4385, 4400, 4410, 4541, **4576**, 4926, 5050	cattle (3) goose (2) turkey (2) duck (1)
3	1	PN-MAC-LA	4488	turkey
3	1	PN-TET-LA	**4483**	duck
3	2	TET-LA-SA	**4319**, 4573	duck, goose
4	6	PN-AG-LA-SA	**4149**, 4174, 4190, 4193, 4199, **4251**	cattle (5) albatross (1)
4	1	PN-CEP-LA-FQ	4148	cattle
4	4	PN-LA-SA-FQ	**4147, 4217, 4231**, **4253**	goose (3) duck (1)
4	2	PN-MAC-LA-SA	4216, **4221**	cattle (1) turkey (1)
4	2	PN-TET-LA-SA	4138, 4218	duck, goose
5	1	PN-AG-LA-SA-FQ	3770	cattle
5	1	PN-MAC-LA-SA-FQ	4122	duck
5	1	PN-TET-LA-SA-FQ	4082	duck
6	1	PN-AG-TET-MAC-LA-SA	3378	cattle
7	1	PN-AG-MAC-LA-SA-FC-FQ	**3036**	cattle
8	1	PN-AG-TET-MAC-LA-SA-FC-FQ	**3029**	cattle

**Table 2:** Distribution of MIC values per antibiotic, including MIC_50_ and MIC_90_, calculated resistance rates, and epidemiological cut-off value (ECOFF) values. Bold vertical lines indicate breakpoints for resistance. Abbreviations: S: susceptible; I: intermediate; R: resistant; PEN: penicillin; AMX: amoxicillin; AMP: ampicillin; FUR: ceftiofur; TTC: tetracycline; DOX: doxycycline; ERY: erythromycin; TIL: tilmicosin; CD: clindamycin; FFC: florfenicol; ENO: enrofloxacin; SMX: sulfamethoxazole; UB: flumequine; APR: apramycin.

	Minimum Inhibitory Concentration (µg/mL)																											MIC_50_	MIC_90_	R (%)	ECOFF (µg/mL)
	512<	512	256<	256	128<	128	64<	64	32<	32	16<	16	8	4	2	1	0.5	0.25	0.25>	0.125	0.125>	0.06	0.06>	0.03	0.03>	0.015	0.015>				
PEN											8	0	1	0	0	1	13	19	0	24	0	11	0	3	0	0	0	0.25 ^S^	8 ^R^	12.5	-
AMX							0	0	0	0	0	4	0	4	5	29	31	6	0	1	0	0	0					1 ^I^	2 ^R^	16.3	0.5
AMP							2	2	0	0	0	0	2	3	1	16	32	9	0	13	0	0	0					0.5 ^S^	4 ^R^	12.5	0.5
FUR											1	0	2	1	4	9	13	13	0	6	0	1	0	19	0	1	10	0.25 ^S^	1 ^S^	3.8	0.06
TTC									0	4	0	2	2	0	0	2	13	38	0	11	0	7	0	1	0			0.25 ^S^	1 ^S^	10.0	2
DOX							0	0	0	0	0	0	2	3	2	1	2	15	0	28	0	11	16					0.125 ^S^	0.25 ^S^	2.5	1
ERY							2	2	0	2	0	3	5	8	25	22	6	4	0	1	0	0	0					2 ^I^	16 ^R^	17.5	16
TIL			1	1	0	0	0	1	0	2	0	3	7	22	23	12	5	0	3									2 ^S^	8 ^S^	7.5	32
CD					6	1	0	12	0	45	0	16	0	0	0	0	0	0	0	0	0							32 ^R^	64 ^R^	100.0	-
FFC							0	0	0	1	0	1	0	1	4	2	45	14	0	12	0	0	0					0.5 ^S^	1 ^S^	2.5	1
ENO											0	0	0	0	4	1	3	3	0	3	0	9	0	14	0	1	42	0.015> ^S^	0.25 ^S^	5.0	0.06
SMX	51	5	0	13	0	8	0	1	0	1	0	1	0	0	0	0	0											512< ^R^	512< ^R^	70.0	-
UB							1	3	0	2	0	1	0	4	2	5	3	9	0	23	0	0	27					0.125	4	-	0.5
APR							1	12	0	33	0	28	5	0	1	0	0	0	0	0	0	0	0					32	64	-	-

**Table 3:** Antibiotic susceptibility profiles and host species of multidrug-resistant strains identified by the broth microdilution method. The strains in bold were also found to be multidrug-resistant by disk diffusion. Abbreviations: PN: penicillins; TET: tetracyclines; MAC: macrolides; LA: lincosamides; SA: sulfonamides; FC: phenicols; FQ: fluoroquinolones; CEP: cephalosporins; *n*: number of bacterial strains.

MIC				
No. of Antibiotic Classes with Resistant Strains	No. of the Resistant Strains	Antibiotic Classes	Strain ID	Host Species (*n*)
3	2	LA-SA-FC	**4319**, **4480**	goose
3	3	MAC-LA-SA	**4217**, 4318, 4924	cattle (1) goose (1) turkey (1)
3	4	PN-LA-SA	**4253,** 4340, **4376**, **4483**	cattle (1) duck (1) goose (1) turkey (1)
3	1	TET-LA-SA	**4380**	duck
4	1	TET-MAC-LA-SA	**4251**	cattle
4	3	PN-MAC-LA-SA	3509, 3699, 3700	cattle (1) goat (2)
4	1	PN-CEP-LA-SA	**4576**	duck
4	1	MAC-LA-FQ-SA	**4147**	cattle
5	1	CEP-TET-MAC-LA-SA	4539	goose
5	1	PN-CEP-LA-FQ-SA	**4149**	cattle
5	3	PN-TET-MAC-LA-SA	**3029, 3036**, **4221**	cattle (1) goose (2)
5	1	TET-MAC-LA-FQ-SA	**4231**	cattle

## Data Availability

Raw sequencing data for the *P. multocida* isolates have been deposited to the NCBI Sequence Read Archive under BioProject ID PRJNA1270947 and PRJNA1288180. (Will be available as soon as the necessary publications are accepted, or by 31 January 2026 at the latest.) Accession numbers are listed in Supplementary Table S1.

## Supplementary Materials

The following supporting information can be downloaded at: https://www.mdpi.com/article/10.3390/antibiotics14090906/s1, Table S1: Metadata of *P. multocida* isolates (*n* = 80) including origin, host species, year of isolation, and BioSample accession numbers; Table S2: Phenotypic and genotypic antibiotic susceptibility profiles of *P. multocida* isolates across B-lactams; Table S3: Phenotypic and genotypic antibiotic susceptibility profiles of *P. multocida* isolates across tetracyclines; Table S4: Phenotypic and genotypic antibiotic susceptibility profiles of *P. multocida* isolates across aminoglycosides; Table S5: Phenotypic and genotypic antibiotic susceptibility profiles of *P. multocida* isolates across phenicols; Table S6: Phenotypic and genotypic antibiotic susceptibility profiles of *P. multocida* isolates across sulfonamids; Table S7: Phenotypic and genotypic antibiotic susceptibility profiles of *P. multocida* isolates across macrolides; Table S8: Phenotypic and genotypic antibiotic susceptibility profiles of *P. multocida* isolates across fluoroquinolones; Table S9: Phenotypic and genotypic antibiotic susceptibility profiles of *P. multocida* isolates across lincosamids.

## Abbreviations

The following abbreviations are used in this manuscript:

ARG	antimicrobial resistance gene
BHI	Brain Heart Infusion
BV-BRC-	Bacterial and Viral Bioinformatics Resource Center
CARD	Comprehensive Antibiotic Resistance Database
CLSI	Clinical and Laboratory Standards Institute
ECOFF	epidemiological cut-off value
EUCAST	European Committee on Antimicrobial Susceptibility Testing
MDR	multidrug-resistant
MIC	minimal inhibitory concentration
PCR	polymerase chain reaction
RGI	Resistance Gene Identifier
SNP	single-nucleotide polymorphism
