# Supplementary material for: Comparative Analysis of Phenotypic and Genotypic Antibiotic Susceptibility of Pasteurella multocida Isolated from Various Host Species in France and Hungary
Source: Antibiotics (Basel). 2025 Sep 8;14(9):906. doi: 10.3390/antibiotics14090906 (PMC12466733; doi:10.3390/antibiotics14090906)
Supplement: Supplementary file 1 [file antibiotics-14-00906-s001.zip › antibiotics-3809681-supplementary.pdf]

Table S1. Metadata of P. multocida isolates (n=80) including origin, host species, year of isolation, and BioSample accession numbers

.

Strain	Host	Collection date	Isolation source	Origin of the strain	Number of reads (Illumina)	Genome size (bp)	Average coverage	GC%	Accession number
3029	goose	2017	heart	Hungary	36,016,201	2,350,680	2295.2	40.9	SAMN48840887
3036	goose	2017	lung	Hungary	13,478,778	2,406,747	870.2	40.6	SAMN48840888
3083	human	2017	wound	Hungary	2,145,372	2,343,919	138.6	40.6	SAMN49835348
3171	dog	2018	nose	Hungary	2,108,082	2,345,429	136.1	40.8	SAMN49835349
3378	cattle	2005	milk	Hungary	3,804,149	2,329,171	241.9	41.2	SAMN48840889
3509	cattle	2007	lung	Hungary	24,188,541	2,341,717	1545.0	40.7	SAMN48840890
3546	sheep	2007	lung	Hungary	4,789,943	2,330,779	305.4	40.5	SAMN49835350
3617	goose	2008	heart	Hungary	5,460,040	2,363,168	353.9	40.4	SAMN48840891
3687	cattle	2008	nose	Hungary	4,581,997	2,329,092	292.7	41.0	SAMN48840892
3699	goat	2008	nose	Hungary	24,980,015	2,341,124	1601.3	41.2	SAMN49835351
3700	goat	2008	lung	Hungary	31,294,145	2,346,708	1996.5	40.6	SAMN49835352
3770	goose	2009	liver	Hungary	5,216,019	2,357,585	337.7	41.0	SAMN48840893
3771	duck	2009	liver	Hungary	2,174,857	2,340,473	140.7	40.8	SAMN48840894
3903	duck	2010	heart	Hungary	1,958,055	2,339,944	126.7	40.8	SAMN48840895
4082	goose	2011	ovarian follicle	Hungary	1,844,984	2,336,149	119.4	40.7	SAMN48840896
4107	duck	2012	n.d.	Hungary	14,648,075	2,373,053	948.1	40.7	SAMN48840897
4117	swine	2014	nose	Hungary	28,866,822	2,345,661	1839.9	40.8	SAMN48840898
4122	cattle	2013	lung	Hungary	3,951,375	2,330,174	251.9	40.5	SAMN48840899
4125	cattle	2013	milk	Hungary	4,330,913	2,328,239	277.4	40.8	SAMN48840900
4138	cattle	2013	lung	Hungary	6,047,237	2,331,015	387.3	40.7	SAMN48840901
4144	cattle	2013	milk	Hungary	4,629,583	2,330,005	294.4	40.7	SAMN48840902
4145	cattle	2013	nose	Hungary	5,317,576	2,332,570	337.4	40.3	SAMN48840903
4147	cattle	2013	milk	Hungary	3,870,957	2,329,251	247.5	40.7	SAMN48840904
4148	cattle	2013	milk	Hungary	5,163,622	2,329,650	329.1	40.8	SAMN48840905
4149	cattle	2013	milk	Hungary	4,137,776	2,330,475	262.4	40.6	SAMN48840906
4162	sheep	2015	lung	Hungary	4,808,676	2,330,091	307.2	40.9	SAMN49835353
4174	cattle	2012	milk	Hungary	4,618,120	2,329,926	294.9	41.1	SAMN48840907
4190	cattle	2015	lung	Hungary	5,409,845	2,330,590	346.3	40.9	SAMN48840908
4193	cattle	2015	fetal stomach	Hungary	4,628,427	2,331,623	290.8	41.2	SAMN48840909
4199	cattle	2015	nose	Hungary	5,354,072	2,329,902	343.1	40.9	SAMN48840910
4201	cattle	2015	lung	Hungary	3,990,137	2,329,164	254.5	40.9	SAMN48840911
4203	cattle	2015	fetal stomach	Hungary	4,214,076	2,330,045	266.5	40.7	SAMN48840912
4216	cattle	2014	nose	Hungary	5,005,029	2,329,575	319.9	40.7	SAMN48840913
4217	cattle	2014	nose	Hungary	4,369,214	2,329,542	278.7	40.9	SAMN48840914
4218	cattle	2014	nose	Hungary	3,885,804	2,329,203	246.8	40.6	SAMN48840915
4221	cattle	2014	lung	Hungary	4,815,431	2,329,741	304.0	40.3	SAMN48840916
4228	rabbit	2015	lung	Hungary	15,955,467	2,366,087	1033.9	40.7	SAMN48840917
4231	cattle	2015	lung	Hungary	4,690,052	2,330,413	296.8	40.9	SAMN48840918
4246	swine	2016	nose	Hungary	4,537,722	2,329,086	290.5	40.8	SAMN49835354
4251	cattle	2016	lung	Hungary	4,561,658	2,329,337	290.0	40.8	SAMN48840919
4252	sheep	2016	lung	Hungary	4,867,153	2,329,273	306.7	40.4	SAMN49835355
4253	cattle	2016	spleen	Hungary	5,326,236	2,330,583	335.1	40.6	SAMN48840920
4254	cattle	2016	spleen	Hungary	6,143,877	2,350,638	397.9	40.8	SAMN48840921
4318	goose	2018	n.d.	Hungary	4,378,488	2,330,984	273.7	40.3	SAMN48840922
4319	goose	2018	n.d.	Hungary	3,594,138	2,329,274	229.1	40.5	SAMN48840923
4330	goat	2016	lung	Hungary	3,957,384	2,328,950	253.0	40.7	SAMN49835356
4340	turkey	2017	n.d.	Hungary	4,541,613	2,329,384	289.0	40.6	SAMN48840924
4341	turkey	2017	n.d.	Hungary	4,301,637	2,328,628	274.9	40.6	SAMN48840925
4373	duck	2015	n.d.	France	8,884,688	2,368,222	571.4	40.8	SAMN48840926
4376	duck	2015	n.d.	France	12,229,741	2,373,678	787.8	40.9	SAMN48840927
4380	duck	2004	n.d.	France	12,443,096	2,369,627	801.1	40.8	SAMN48840928
4385	duck	2015	n.d.	France	11,878,364	2,414,753	764.0	40.4	SAMN48840929

4389	turkey	2016	n.d.	France	5,758,017	2,350,007	372.7	40.6	SAMN48840930
4390	turkey	2017	n.d.	France	6,598,046	2,359,320	427.2	40.6	SAMN48840931
4400	turkey	2018	n.d.	France	11,517,066	2,361,551	741.4	40.6	SAMN48840932
4410	turkey	2017	n.d.	France	6,364,802	2,349,661	412.4	40.8	SAMN48840933
4473	goose	2018	n.d.	Hungary	4,889,182	2,365,602	316.2	40.9	SAMN48840934
4480	goose	2018	n.d.	Hungary	12,078,371	2,360,690	777.9	40.6	SAMN48840935
4483	goose	2018	n.d.	Hungary	11,407,463	2,361,026	734.5	40.6	SAMN48840936
4488	duck	2017	n.d.	France	11,346,745	2,342,961	730.8	40.7	SAMN48840937
4489	duck	2017	n.d.	France	12,131,941	2,372,567	781.8	40.5	SAMN48840938
4492	turkey	2017	n.d.	France	15,833,231	2,440,217	1025.4	40.4	SAMN48840939
4499	albatross	2019	n.d.	France	15,878,917	2,399,554	1030.5	40.5	SAMN49835357
4502	albatross	2019	n.d.	France	22,239,143	2,441,004	1451.0	40.5	SAMN49835358
4539	goose	2019	liver	Hungary	15,116,100	2,402,327	978.9	40.5	SAMN48840940
4541	goose	2019	n.d.	Hungary	2,444,644	2,349,976	158.1	40.8	SAMN48840941
4542	goose	2019	n.d.	Hungary	5,438,426	2,367,830	351.9	40.7	SAMN48840942
4556	goose	2019	n.d.	Hungary	14,769,564	2,373,704	960.6	40.7	SAMN48840943
4564	goose	2019	n.d.	Hungary	10,825,888	2,428,639	693.9	40.4	SAMN48840944
4565	goose	2019	n.d.	Hungary	12,613,185	2,463,323	807.2	40.3	SAMN48840945
4573	goose	2020	n.d.	Hungary	12,071,211	2,413,991	775.8	40.6	SAMN48840946
4574	goose	2020	n.d.	Hungary	10,865,912	2,403,741	698.3	40.6	SAMN48840947
4576	duck	2020	n.d.	Hungary	8,690,161	2,380,226	558.1	40.7	SAMN48840948
4586	fallow deer	2020	spleen	Hungary	13,506,672	2,483,841	865.7	40.6	SAMN49835359
4674	turkey	2021	n.d.	Hungary	9,348,153	2,399,775	599.4	40.3	SAMN48840949
4772	albatross	2019	n.d.	France	9,024,285	2,365,930	584.6	40.4	SAMN49835360
4924	turkey	2019	n.d.	France	9,307,981	2,349,863	599.1	40.8	SAMN48840950
4926	turkey	2022	lung	Hungary	16,305,168	2,385,383	1050.0	41.1	SAMN48840951
5050	turkey	2023	n.d.	France	15,335,597	2,364,623	993.7	40.7	SAMN48840952
5144	swine	2023	nose	Hungary	5,712,933	2,391,710	368.8	41.2	SAMN49835361

n.d.: no data.

Table S2. Phenotypic and genotypic antibiotic susceptibility profiles of P. multocida isolates across B-lactams.

Strain ID	Country	Host species	ß-lactams: penicillins & cephalosporins											
			Disk					MIC				ARG		
			PEN	AMX	AMP	FUR	CFX	PEN (0.015 - 16 µg/mL)	AMX (0.06 - 64 µg/mL)	AMP (0.06 - 64 µg/mL)	FUR (0.015 - 16 µg/mL)	*bla*-ROB1	*bla*-TEM	*bla*-OXA-2
3029	Hungary	goose	R	S	S	S	S	0.5	0.5	4	0.5	-	-	-
3036	Hungary	goose	S	S	S	S	S	1	2	1	1	-	-	-
3083	Hungary	human	S	S	S	S	S	0.03	0.25	0.125	0.03	-	-	-
3171	Hungary	dog	S	S	S	S	S	0.06	0.25	0.125	0.03	-	-	-
3378	Hungary	cattle	S	S	S	S	S	0.25	0.5	0.5	0.015	-	-	-
3509	Hungary	cattle	S	S	S	S	S	16 <	4	8	1	-	-	-
3546	Hungary	sheep	S	S	S	S	S	0.125	0.25	0.125	0.015 >	-	-	-
3617	Hungary	goose	S	S	S	S	S	0.06	0.5	0.125	0.03	-	-	-
3687	Hungary	cattle	S	S	S	S	S	0.25	1	1	0.25	-	-	-
3699	Hungary	goat	S	S	S	S	S	16 <	4	8	1	-	-	-
3700	Hungary	goat	S	S	S	S	S	16 <	4	4	1	-	-	-
3770	Hungary	goose	S	R	S	S	S	0.06	0.5	0.125	0.03	-	-	-
3771	Hungary	duck	S	S	S	S	S	0.06	0.5	0.125	0.03	-	-	-
3903	Hungary	duck	S	S	S	S	S	0.06	0.25	0.125	0.03	-	-	-
4082	Hungary	goose	S	R	R	S	S	0.06	0.25	0.125	0.03	-	-	-
4107	Hungary	duck	S	S	R	S	S	0.06	0.5	0.125	0.03	-	-	-
4117	Hungary	swine	S	S	S	S	S	0.25	0.5	1	0.25	-	-	-
4122	Hungary	cattle	S	S	S	S	S	0.25	0.5	0.25	0.03	-	-	-
4125	Hungary	cattle	S	S	S	S	S	0.25	0.5	0.5	0.03	-	-	-
4138	Hungary	cattle	S	S	R	S	S	16 <	4	4	2	-	-	-
4144	Hungary	cattle	S	S	S	S	S	0.5	1	0.5	0.03	-	-	-
4145	Hungary	cattle	S	S	S	S	S	0.03	0.5	0.5	0.125	-	-	-
4147	Hungary	cattle	S	S	R	S	S	0.125	1	0.5	0.25	-	-	-
4148	Hungary	cattle	S	S	S	S	S	0.5	0.5	1	0.5	-	-	-
4149	Hungary	cattle	S	S	R	R	I	16 <	16	64	16 <	-	-	-
4162	Hungary	sheep	S	S	S	S	S	0.25	0.5	1	0.015 >	-	-	-
4174	Hungary	cattle	S	R	R	S	S	0.5	0.5	0.5	0.015 >	-	-	-
4190	Hungary	cattle	R	R	R	S	S	0.25	0.5	0.5	0.125	-	-	-
4193	Hungary	cattle	S	R	R	S	S	0.125	1	0.5	0.25	-	-	-
4199	Hungary	cattle	R	R	R	S	S	0.5	1	1	0.5	-	-	-
4201	Hungary	cattle	S	S	R	S	S	0.125	1	0.25	0.06	-	-	-
4203	Hungary	cattle	S	S	R	S	S	0.125	0.5	0.25	0.25	-	-	-
4216	Hungary	cattle	S	S	R	S	S	0.25	1	1	0.03	-	-	-
4217	Hungary	cattle	S	S	R	S	S	0.5	0.5	0.5	0.125	-	-	-
4218	Hungary	cattle	S	S	R	S	S	0.5	1	1	0.015 >	-	-	-
4221	Hungary	cattle	R	R	R	S	S	16 <	16	64	2	+	-	+
4228	Hungary	rabbit	S	S	S	S	S	0.03	0.125	0.125	0.03	-	-	-
4231	Hungary	cattle	R	R	R	S	S	0.5	0.5	1	1	-	-	-
4246	Hungary	swine	S	S	S	S	S	0.25	0.25	0.25	0.25	-	-	-
4251	Hungary	cattle	S	R	R	S	S	0.125	1	1	0.03	-	-	-
4252	Hungary	sheep	S	R	S	S	S	0.25	1	0.5	0.015 >	-	-	-
4253	Hungary	cattle	R	R	R	S	S	0.5	2	0.5	4	-	-	-
4254	Hungary	cattle	S	S	S	S	S	0.06	0.5	0.125	0.03	-	-	-
4318	Hungary	goose	S	S	S	S	S	0.06	0.5	0.25	0.5	-	-	-
4319	Hungary	goose	S	S	S	S	S	0.125	0.5	0.5	0.5	-	-	-
4330	Hungary	goat	S	S	S	S	S	0.25	0.5	0.25	0.125	-	-	-
4340	Hungary	turkey	S	S	S	S	S	0.125	2	2	1	-	-	-
4341	Hungary	turkey	S	S	S	S	S	0.125	0.5	0.25	0.5	-	-	-
4373	France	duck	S	S	R	S	S	0.25	1	1	0.5	-	-	-
4376	France	duck	S	S	R	I	S	8	0.5	0.5	4	-	-	-
4380	France	duck	S	S	R	S	S	0.06	0.5	0.25	0.25	-	-	-

4385	France	duck	S	S	R	S	S	0.25	1	1	0.5	-	-	-
4389	France	turkey	R	S	S	S	S	0.125	0.5	0.5	0.5	-	-	-
4390	France	turkey	S	S	S	S	S	0.125	0.5	0.5	0.125	-	-	-
4400	France	turkey	S	S	S	S	S	0.125	1	0.5	0.5	-	-	-
4410	France	turkey	R	R	R	S	S	0.25	1	1	0.5	-	-	-
4473	Hungary	goose	S	S	S	S	S	0.125	1	0.5	0.25	-	-	-
4480	Hungary	goose	S	S	S	S	S	0.125	1	0.5	0.25	-	-	-
4483	Hungary	goose	R	R	R	S	S	16 <	16	64 <	1	+	-	-
4488	France	duck	R	R	S	S	S	0.125	1	0.5	0.5	-	-	-
4489	France	duck	S	S	S	S	S	0.125	1	0.5	1	-	-	-
4492	France	turkey	S	S	S	S	S	0.25	1	1	0.25	-	-	-
4499	France	albatross	S	S	S	S	S	0.125	1	0.5	0.125	-	-	-
4502	France	albatross	S	R	S	S	S	0.125	1	0.5	0.25	-	-	-
4539	Hungary	goose	S	S	S	S	S	0.25	1	0.5	8	-	-	-
4541	Hungary	goose	S	R	S	S	S	0.125	0.5	0.5	0.25	-	-	-
4542	Hungary	goose	S	S	S	S	S	0.25	1	0.5	1	-	-	-
4556	Hungary	goose	S	S	S	S	S	0.125	0.5	0.5	0.25	-	-	-
4564	Hungary	goose	S	S	S	S	S	0.125	1	0.5	0.03	-	-	-
4565	Hungary	goose	S	S	S	S	S	0.125	1	0.5	0.015 >	-	-	-
4573	Hungary	goose	R	S	S	S	S	0.5	1	0.5	2	-	-	-
4574	Hungary	goose	S	S	S	S	S	0.25	1	1	2	-	-	-
4576	Hungary	duck	R	R	R	S	S	16 <	16	64 <	8	+	-	-
4586	Hungary	fallow deer	S	S	S	S	S	0.5	2	0.5	0.015 >	-	-	-
4674	Hungary	turkey	S	S	S	S	S	0.25	1	1	0.5	-	-	-
4772	France	albatross	S	S	S	S	S	0.125	1	0.5	0.015 >	-	-	-
4924	France	turkey	S	S	S	S	S	0.125	0.5	0.25	0.015 >	-	-	-
4926	Hungary	turkey	R	R	R	S	S	0.5	2	0.5	0.015 >	-	-	-
5050	France	turkey	S	R	S	S	S	0.25	0.5	0.125	0.03	-	-	-
5144	Hungary	swine	S	S	S	S	S	0.06	0.5	0.125	0.03	-	-	-
	Resistance %		16.25	23.75	32.5	1.25	0	12.5	16.25	12.5	3.75	3.75	0	1.25

The last row shows the percentage of resistance detected in each antibiotic. Abbreviation: PEN: penicillin; AMX: amoxicillin; AMP: ampicillin; FUR: ceftiofur; CFX: cephalexin; GEN: gentamicin; TTC: tetracycline; DOX: doxycycline; ERY: erythromycin; CD: clindamycin; FFC: florfenicol; CHL: chloramphenicol; SMX: sulfamethoxazole; CPR: ciprofloxacin; ENO: enrofloxacin; UB: flumequine; TIL: tilmicosin; ARG: antibiotic-resistant gene; S: susceptible; I: intermediate; R: resistant; +: present; MIC: minimal inhibitory concentration; SNP: single-nucleotide polymorphism; ARG: antimicrobial resistance gene; AA: amino acid.

Table S3. Phenotypic and genotypic antibiotic susceptibility profiles of P. multocida isolates across tetracyclines.

Strain ID	Country	Host species	Tetracyclines					
			Disk		MIC		ARG	
			TTC	DOX	TTC (0.03 - 32 µg/mL)	DOX (0.06 - 64 µg/mL)	*tet*B	*tet*H
3029	Hungary	goose	R	R	32	4	-	+
3036	Hungary	goose	R	R	32	8	-	+
3083	Hungary	human	S	S	0.25	0.06	-	-
3171	Hungary	dog	S	S	0.25	0.06	-	-
3378	Hungary	cattle	S	S	0.25	0.125	-	-
3509	Hungary	cattle	S	S	0.25	0.25	-	-
3546	Hungary	sheep	S	S	0.125	0.06 >	-	-
3617	Hungary	goose	S	S	0.125	0.06	-	-
3687	Hungary	cattle	S	S	0.5	0.25	-	-
3699	Hungary	goat	S	S	0.25	0.125	-	-
3700	Hungary	goat	S	S	0.25	0.125	-	-
3770	Hungary	goose	S	S	0.25	0.06	-	-
3771	Hungary	duck	S	S	0.25	0.06	-	-
3903	Hungary	duck	S	S	0.25	0.06	-	-

4082	Hungary	goose	S	S	0.25	0.06	-	-
4107	Hungary	duck	S	S	0.25	0.06	-	-
4117	Hungary	swine	S	S	0.5	0.125	-	-
4122	Hungary	cattle	S	S	1	0.5	-	-
4125	Hungary	cattle	S	S	0.5	0.25	-	-
4138	Hungary	cattle	S	S	0.25	0.25	-	-
4144	Hungary	cattle	S	S	0.25	0.06 >	-	-
4145	Hungary	cattle	S	S	0.125	0.25	-	-
4147	Hungary	cattle	S	S	0.5	0.25	-	-
4148	Hungary	cattle	S	S	0.25	0.125	-	-
4149	Hungary	cattle	S	S	0.5	0.5	-	-
4162	Hungary	sheep	S	S	0.25	0.125	-	-
4174	Hungary	cattle	S	S	0.5	0.125	-	-
4190	Hungary	cattle	S	S	0.25	0.125	-	-
4193	Hungary	cattle	S	S	0.25	0.125	-	-
4199	Hungary	cattle	S	S	0.125	0.06 >	-	-
4201	Hungary	cattle	S	S	0.5	0.25	-	-
4203	Hungary	cattle	S	S	0.25	0.125	-	-
4216	Hungary	cattle	S	S	0.25	0.125	-	-
4217	Hungary	cattle	S	S	0.25	0.06 >	-	-
4218	Hungary	cattle	S	S	0.25	0.06 >	-	-
4221	Hungary	cattle	R	R	8	1	-	+
4228	Hungary	rabbit	S	S	0.125	0.06	-	-
4231	Hungary	cattle	I	I	16	2	+	-
4246	Hungary	swine	S	S	0.125	0.06 >	-	-
4251	Hungary	cattle	R	R	16	4	+	-
4252	Hungary	sheep	S	S	0.06	0.125	-	-
4253	Hungary	cattle	S	S	0.5	0.25	-	-
4254	Hungary	cattle	S	S	0.25	0.06	-	-
4318	Hungary	goose	S	S	0.25	0.125	-	-
4319	Hungary	goose	S	S	0.25	0.125	-	-
4330	Hungary	goat	S	S	0.06	0.25	-	-
4340	Hungary	turkey	S	S	0.06	0.06 >	-	-
4341	Hungary	turkey	S	S	0.03	0.06 >	-	-
4373	France	duck	R	S	0.06	0.06 >	-	-
4376	France	duck	S	S	0.06	0.125	-	-
4380	France	duck	R	R	8	4	-	+
4385	France	duck	S	S	0.06	0.125	-	-
4389	France	turkey	S	I	0.125	0.25	-	-
4390	France	turkey	S	S	0.06	0.125	-	-
4400	France	turkey	S	S	0.5	0.125	-	-
4410	France	turkey	S	S	0.5	0.25	-	-
4473	Hungary	goose	S	S	0.25	0.125	-	-
4480	Hungary	goose	S	S	0.25	0.125	-	-
4483	Hungary	goose	S	S	0.25	0.125	-	-
4488	France	duck	S	S	0.5	0.25	-	-
4489	France	duck	R	R	32	8	-	+
4492	France	turkey	S	S	1	0.125	-	-
4499	France	albatross	S	S	0.125	0.06 >	-	-
4502	France	albatross	S	S	0.25	0.125	-	-
4539	Hungary	goose	S	S	32	2	-	-
4541	Hungary	goose	S	S	0.5	0.25	-	-
4542	Hungary	goose	S	S	0.25	0.06 >	-	-
4556	Hungary	goose	S	S	0.25	0.06 >	-	-
4564	Hungary	goose	S	S	0.25	0.125	-	-
4565	Hungary	goose	S	S	0.25	0.06 >	-	-
4573	Hungary	goose	S	S	0.25	0.06 >	-	-
4574	Hungary	goose	S	S	0.5	0.125	-	-
4576	Hungary	duck	S	S	0.25	0.125	-	-
4586	Hungary	fallow deer	S	S	0.125	0.06>	-	-

4674	Hungary	turkey	S	S	0.25	0.125	-	-
4772	France	albatross	S	S	0.125	0.06 >	-	+
4924	France	turkey	S	S	0.25	0.25	-	-
4926	Hungary	turkey	S	S	0.125	0.125	-	-
5050	France	turkey	S	S	0.25	0.25	-	-
5144	Hungary	swine	S	S	0.25	0.06	-	-
	Resistance %		8.8	7.5	10.0	2.5	2.5	7.5

The last row shows the percentage of resistance detected in each antibiotic. Abbreviation: PEN: penicillin; AMX: amoxicillin; AMP: ampicillin; FUR: ceftiofur; CFX: cephalexin; GEN: gentamicin; TTC: tetracycline; DOX: doxycycline; ERY: erythromycin; CD: clindamycin; FFC: florfenicol; CHL: chloramphenicol; SMX: sulfamethoxazole; CPR: ciprofloxacin; ENO: enrofloxacin; UB: flumequine; TIL: tilmicosin; ARG: antibiotic-resistant gene; S: susceptible; I: intermediate; R: resistant; +: present; MIC: minimal inhibitory concentration; SNP: single-nucleotide polymorphism; ARG: antimicrobial resistance gene; AA: amino acid.

Table S4. Phenotypic and genotypic antibiotic susceptibility profiles of P. multocida isolates across aminoglycosides.

Strain ID	Country	Host species	Aminoglycosides						
			Disk	MIC	ARG				
			GEN	APR (0.06 - 64 µg/mL)	*str*A	*aph*A1	*str*B	*aad*B	*aad*A25
3029	Hungary	goose	I	16	-	-	-	-	-
3036	Hungary	goose	I	8	-	-	-	-	-
3083	Hungary	human	S	16	-	-	-	-	-
3171	Hungary	dog	S	16	-	-	-	-	-
3378	Hungary	cattle	R	32	-	-	-	-	-
3509	Hungary	cattle	I	8	-	-	-	-	-
3546	Hungary	sheep	S	2	-	-	-	-	-
3617	Hungary	goose	S	32	-	-	-	-	-
3687	Hungary	cattle	S	16	-	-	-	-	-
3699	Hungary	goat	S	64	-	-	-	-	-
3700	Hungary	goat	S	8	-	-	-	-	-
3770	Hungary	goose	S	32	-	-	-	-	-
3771	Hungary	duck	S	32	-	-	-	-	-
3903	Hungary	duck	S	16	-	-	-	-	-
4082	Hungary	goose	I	32	-	-	-	-	-
4107	Hungary	duck	I	32	+	-	-	-	-
4117	Hungary	swine	S	32	-	-	-	-	-
4122	Hungary	cattle	R	32	-	-	-	-	-
4125	Hungary	cattle	I	8	-	-	-	-	-
4138	Hungary	cattle	I	64	-	-	-	-	-
4144	Hungary	cattle	S	32	-	-	-	-	-
4145	Hungary	cattle	I	32	-	-	-	-	-
4147	Hungary	cattle	R	32	-	-	-	-	-
4148	Hungary	cattle	R	64	-	-	-	-	-
4149	Hungary	cattle	S	32	-	-	-	-	-
4162	Hungary	sheep	S	16	-	-	-	-	-
4174	Hungary	cattle	R	64 <	-	-	-	-	-
4190	Hungary	cattle	R	64	-	-	-	-	-
4193	Hungary	cattle	I	64	-	-	-	-	-
4199	Hungary	cattle	R	32	-	-	-	-	-
4201	Hungary	cattle	I	16	-	-	-	-	-
4203	Hungary	cattle	I	64	-	-	-	-	-
4216	Hungary	cattle	R	64	-	-	-	-	-
4217	Hungary	cattle	R	64	-	-	-	-	-
4218	Hungary	cattle	I	16	-	-	-	-	-
4221	Hungary	cattle	R	64	+	+	+	+	+
4228	Hungary	rabbit	S	16	-	-	-	-	-

4231	Hungary	cattle	R	32	+	-	-	-	-
4246	Hungary	swine	S	16	-	-	-	-	-
4251	Hungary	cattle	R	16	+	-	-	-	-
4252	Hungary	sheep	I	16	-	-	-	-	-
4253	Hungary	cattle	I	16	-	-	-	-	-
4254	Hungary	cattle	I	16	-	-	-	-	-
4318	Hungary	goose	I	32	-	-	-	-	-
4319	Hungary	goose	S	64	-	-	-	-	-
4330	Hungary	goat	S	16	-	-	-	-	-
4340	Hungary	turkey	S	16	-	-	-	-	-
4341	Hungary	turkey	S	16	-	-	-	-	-
4373	France	duck	S	32	+	-	+	-	-
4376	France	duck	S	32	-	-	-	-	-
4380	France	duck	S	32	-	-	-	-	-
4385	France	duck	S	32	-	-	-	-	-
4389	France	turkey	S	32	-	-	-	-	-
4390	France	turkey	I	64	-	-	-	-	-
4400	France	turkey	S	32	-	-	-	-	-
4410	France	turkey	S	64	-	-	-	-	-
4473	Hungary	goose	S	32	-	-	-	-	-
4480	Hungary	goose	S	32	-	-	-	-	-
4483	Hungary	goose	I	32	+	-	-	-	-
4488	France	duck	I	32	-	-	-	-	-
4489	France	duck	I	32	-	-	-	-	-
4492	France	turkey	S	32	-	-	-	-	-
4499	France	albatross	S	32	-	-	-	-	-
4502	France	albatross	R	32	-	-	-	-	-
4539	Hungary	goose	S	16	-	-	-	-	-
4541	Hungary	goose	S	16	-	-	-	-	-
4542	Hungary	goose	S	32	-	-	-	-	-
4556	Hungary	goose	S	16	-	-	-	-	-
4564	Hungary	goose	S	8	-	-	-	-	-
4565	Hungary	goose	S	16	-	-	-	-	-
4573	Hungary	goose	S	16	-	-	-	-	-
4574	Hungary	goose	S	32	-	-	-	-	-
4576	Hungary	duck	S	32	-	-	-	-	-
4586	Hungary	fallow deer	S	16	-	-	-	-	-
4674	Hungary	turkey	S	32	-	-	-	-	-
4772	France	albatross	S	16	-	-	-	-	-
4924	France	turkey	S	16	+	-	+	-	-
4926	Hungary	turkey	I	16	-	-	-	-	-
5050	France	turkey	I	16	-	-	-	-	-
5144	Hungary	swine	S	16	-	-	-	-	-
	Resistance %		16.25	-	8.75	1.25	3.75	1.25	1.25

The last row shows the percentage of resistance detected in each antibiotic. Abbreviation: PEN: penicillin; AMX: amoxicillin; AMP: ampicillin; FUR: ceftiofur; CFX: cephalexin; GEN: gentamicin; TTC: tetracycline; DOX: doxycycline; ERY: erythromycin; CD: clindamycin; FFC: florfenicol; CHL: chloramphenicol; SMX: sulfamethoxazole; CPR: ciprofloxacin; ENO: enrofloxacin; UB: flumequine; TIL: tilmicosin; ARG: antibiotic-resistant gene; S: susceptible; I: intermediate; R: resistant; +: present; MIC: minimal inhibitory concentration; SNP: single-nucleotide polymorphism; ARG: antimicrobial resistance gene; AA: amino acid.

Table S5. Phenotypic and genotypic antibiotic susceptibility profiles of P. multocida isolates across phenicols.

**Strain ID**	**Country**	**Host species**	Phenicols				
			Disk		MIC	ARG	
			FFC	CHL	FFC (0.06 - 64 µg/mL)	*catAIII*	*flo*R
3029	Hungary	goose	S	S	0.25	-	-

3036	Hungary	goose	S	S	0.5	-	-
3083	Hungary	human	S	S	0.125	-	-
3171	Hungary	dog	S	S	0.125	-	-
3378	Hungary	cattle	S	S	0.25	-	-
3509	Hungary	cattle	S	S	2	-	-
3546	Hungary	sheep	S	S	0.25	-	-
3617	Hungary	goose	S	S	0.125	-	-
3687	Hungary	cattle	S	S	0.5	-	-
3699	Hungary	goat	S	S	4	-	-
3700	Hungary	goat	S	S	2	-	-
3770	Hungary	goose	S	S	0.125	-	-
3771	Hungary	duck	S	S	0.125	-	-
3903	Hungary	duck	S	S	0.125	-	-
4082	Hungary	goose	S	S	0.125	-	-
4107	Hungary	duck	S	S	0.125	-	-
4117	Hungary	swine	S	S	0.5	-	-
4122	Hungary	cattle	S	S	0.25	-	-
4125	Hungary	cattle	S	S	0.25	-	-
4138	Hungary	cattle	S	S	0.5	-	-
4144	Hungary	cattle	S	S	0.5	-	-
4145	Hungary	cattle	S	S	0.5	-	-
4147	Hungary	cattle	S	S	0.5	-	-
4148	Hungary	cattle	S	S	0.5	-	-
4149	Hungary	cattle	S	S	2	-	-
4162	Hungary	sheep	S	S	0.5	-	-
4174	Hungary	cattle	S	S	0.5	-	-
4190	Hungary	cattle	S	S	0.5	-	-
4193	Hungary	cattle	S	S	0.5	-	-
4199	Hungary	cattle	S	S	1	-	-
4201	Hungary	cattle	S	S	0.5	-	-
4203	Hungary	cattle	S	S	0.5	-	-
4216	Hungary	cattle	S	S	0.5	-	-
4217	Hungary	cattle	S	S	0.5	-	-
4218	Hungary	cattle	S	S	0.25	-	-
4221	Hungary	cattle	S	S	0.5	-	-
4228	Hungary	rabbit	S	S	0.125	-	-
4231	Hungary	cattle	S	R	0.5	+	-
4246	Hungary	swine	S	S	0.5	-	-
4251	Hungary	cattle	S	R	0.5	+	-
4252	Hungary	sheep	S	S	0.5	-	-
4253	Hungary	cattle	S	S	0.5	-	-
4254	Hungary	cattle	S	S	0.125	-	-
4318	Hungary	goose	S	S	0.5	-	-
4319	Hungary	goose	S	R	32	-	+
4330	Hungary	goat	S	S	0.25	-	-
4340	Hungary	turkey	S	S	0.25	-	-
4341	Hungary	turkey	S	S	0.25	-	-
4373	France	duck	S	S	0.5	-	-
4376	France	duck	S	S	0.5	-	-
4380	France	duck	S	S	0.5	-	-
4385	France	duck	S	S	0.5	-	-
4389	France	turkey	S	S	0.25	-	-
4390	France	turkey	I	S	0.25	-	-
4400	France	turkey	S	S	0.5	-	-
4410	France	turkey	S	S	0.5	-	-
4473	Hungary	goose	S	S	0.5	-	-
4480	Hungary	goose	I	I	16	-	+
4483	Hungary	goose	S	S	0.5	-	-
4488	France	duck	S	S	0.5	-	-
4489	France	duck	S	S	0.5	-	-

4492	France	turkey	S	S	0.5	-	-
4499	France	albatross	S	S	0.5	-	-
4502	France	albatross	S	S	0.5	-	-
4539	Hungary	goose	S	S	2	-	-
4541	Hungary	goose	S	S	0.5	-	-
4542	Hungary	goose	S	S	0.25	-	-
4556	Hungary	goose	S	S	0.25	-	-
4564	Hungary	goose	S	S	0.5	-	-
4565	Hungary	goose	S	S	0.5	-	-
4573	Hungary	goose	S	S	0.5	-	-
4574	Hungary	goose	S	S	0.5	-	-
4576	Hungary	duck	S	S	0.5	-	-
4586	Hungary	fallow deer	S	S	0.5	-	-
4674	Hungary	turkey	S	S	1	-	-
4772	France	albatross	S	S	0.25	-	-
4924	France	turkey	S	S	0.5	-	-
4926	Hungary	turkey	S	S	0.5	-	-
5050	France	turkey	S	S	0.125	-	-
5144	Hungary	swine	S	S	0.125	-	-
	Resistance %		0.0	3.75	2.5	2.5	2.5

The last row shows the percentage of resistance detected in each antibiotic. Abbreviation: PEN: penicillin; AMX: amoxicillin; AMP: ampicillin; FUR: ceftiofur; CFX: cephalexin; GEN: gentamicin; TTC: tetracycline; DOX: doxycycline; ERY: erythromycin; CD: clindamycin; FFC: florfenicol; CHL: chloramphenicol; SMX: sulfamethoxazole; CPR: ciprofloxacin; ENO: enrofloxacin; UB: flumequine; TIL: tilmicosin; ARG: antibiotic-resistant gene; S: susceptible; I: intermediate; R: resistant; +: present; MIC: minimal inhibitory concentration; SNP: single-nucleotide polymorphisms; ARG: antimicrobial resistance gene; AA: amino acid.

Table S6. Phenotypic and genotypic antibiotic susceptibility profiles of P. multocida isolates across sulfonamids.

Strain ID	Country	Host species	Sulfonamides			
			Disk	MIC	ARG	
			SMX	SMX (0.5 - 512 µg/mL)	*sul*2	*sul*1
3029	Hungary	goose	R	512 <	-	-
3036	Hungary	goose	R	512 <	-	-
3083	Hungary	human	R	256	-	-
3171	Hungary	dog	I	512	-	-
3378	Hungary	cattle	R	512 <	-	-
3509	Hungary	cattle	I	512 <	-	-
3546	Hungary	sheep	S	256	-	-
3617	Hungary	goose	R	512	-	-
3687	Hungary	cattle	I	256	-	-
3699	Hungary	goat	R	512 <	-	-
3700	Hungary	goat	S	512 <	-	-
3770	Hungary	goose	R	256	-	-
3771	Hungary	duck	R	512	-	-
3903	Hungary	duck	I	128	-	-
4082	Hungary	goose	R	128	-	-
4107	Hungary	duck	I	64	-	-
4117	Hungary	swine	R	512 <	-	-
4122	Hungary	cattle	R	512 <	-	-
4125	Hungary	cattle	R	256	-	-
4138	Hungary	cattle	R	256	-	-
4144	Hungary	cattle	R	512 <	-	-
4145	Hungary	cattle	R	16	-	-
4147	Hungary	cattle	R	512 <	-	-
4148	Hungary	cattle	R	256	-	-
4149	Hungary	cattle	I	512 <	-	-

4162	Hungary	sheep	S	512 <	-	-
4174	Hungary	cattle	R	512 <	-	-
4190	Hungary	cattle	R	256	-	-
4193	Hungary	cattle	R	512 <	-	-
4199	Hungary	cattle	R	256	-	-
4201	Hungary	cattle	S	512 <	-	-
4203	Hungary	cattle	S	512 <	-	-
4216	Hungary	cattle	R	512 <	-	-
4217	Hungary	cattle	R	512 <	-	-
4218	Hungary	cattle	R	128	-	-
4221	Hungary	cattle	R	512 <	+	-
4228	Hungary	rabbit	S	512	-	-
4231	Hungary	cattle	R	512 <	+	-
4246	Hungary	swine	I	128	-	-
4251	Hungary	cattle	R	512	+	-
4252	Hungary	sheep	I	32	-	-
4253	Hungary	cattle	R	512 <	-	-
4254	Hungary	cattle	R	512 <	-	-
4318	Hungary	goose	R	512 <	-	-
4319	Hungary	goose	R	512 <	-	-
4330	Hungary	goat	R	512 <	-	-
4340	Hungary	turkey	S	512 <	-	-
4341	Hungary	turkey	S	512 <	-	-
4373	France	duck	R	512 <	-	+
4376	France	duck	R	512 <	-	+
4380	France	duck	R	512 <	-	-
4385	France	duck	R	512 <	-	-
4389	France	turkey	I	512 <	-	-
4390	France	turkey	R	512 <	-	-
4400	France	turkey	R	512 <	-	-
4410	France	turkey	R	512 <	-	-
4473	Hungary	goose	R	512 <	-	-
4480	Hungary	goose	R	512 <	-	-
4483	Hungary	goose	R	512 <	+	-
4488	France	duck	R	512 <	-	-
4489	France	duck	R	512 <	-	-
4492	France	turkey	R	512 <	-	-
4499	France	albatross	R	512 <	-	-
4502	France	albatross	R	512 <	-	-
4539	Hungary	goose	R	512 <	-	-
4541	Hungary	goose	R	512 <	-	-
4542	Hungary	goose	R	512 <	-	-
4556	Hungary	goose	S	256	-	-
4564	Hungary	goose	R	256	-	-
4565	Hungary	goose	R	256	-	-
4573	Hungary	goose	R	512 <	-	-
4574	Hungary	goose	R	512 <	-	-
4576	Hungary	duck	R	512 <	+	-
4586	Hungary	fallow deer	I	256	-	-
4674	Hungary	turkey	R	512 <	-	-
4772	France	albatross	R	128	-	-
4924	France	turkey	R	512 <	+	-
4926	Hungary	turkey	R	128	-	-
5050	France	turkey	R	128	-	-
5144	Hungary	swine	S	128	-	-
Resistance %			75.0	70.0	7.5	2.5

The last row shows the percentage of resistance detected in each antibiotic. Abbreviation: PEN: penicillin; AMX: amoxicillin; AMP: ampicillin; FUR: ceftiofur; CFX: cephalexin; GEN: gentamicin; TTC: tetracycline; DOX: doxycycline; ERY: erythromycin; CD: clindamycin; FFC: florfenicol; CHL:

chloramphenicol; SMX: sulfamethoxazole; CPR: ciprofloxacin; ENO: enrofloxacin; UB: flumequine; TIL: tilmicosin; ARG: antibiotic-resistant gene; S: susceptible; I: intermediate; R: resistant; +: present; MIC: minimal inhibitory concentration; SNP: single-nucleotide polymorphisms; ARG: antimicrobial resistance gene; AA: amino acid.

Table S7. Phenotypic and genotypic antibiotic susceptibility profiles of P. multocida isolates across macrolides.

Strain ID	Country	Host species	Macrolides					
			Disk	MIC		ARG		
			ERY	ERY (0.06 - 64 μg/mL)	TIL (0.25 - 256 μg/mL)	*erm(42)*	*msr*E	*mph*E
3029	Hungary	goose	I	8	8	-	-	-
3036	Hungary	goose	I	8	16	-	-	-
3083	Hungary	human	I	1	2	-	-	-
3171	Hungary	dog	I	1	1	-	-	-
3378	Hungary	cattle	I	2	4	-	-	-
3509	Hungary	cattle	I	16	32	-	-	-
3546	Hungary	sheep	S	1	1	-	-	-
3617	Hungary	goose	S	1	2	-	-	-
3687	Hungary	cattle	R	64	256	-	-	-
3699	Hungary	goat	I	16	8	-	-	-
3700	Hungary	goat	I	32	8	-	-	-
3770	Hungary	goose	I	1	1	-	-	-
3771	Hungary	duck	I	0.5	1	-	-	-
3903	Hungary	duck	I	2	2	-	-	-
4082	Hungary	goose	I	1	2	-	-	-
4107	Hungary	duck	I	0.25	1	-	-	-
4117	Hungary	swine	S	2	2	-	-	-
4122	Hungary	cattle	S	0.25	0.5	-	-	-
4125	Hungary	cattle	S	0.25	0.25 >	-	-	-
4138	Hungary	cattle	I	4	4	-	-	-
4144	Hungary	cattle	I	0.5	0.25 >	-	-	-
4145	Hungary	cattle	I	0.125	2	-	-	-
4147	Hungary	cattle	I	8	4	-	-	-
4148	Hungary	cattle	I	2	4	-	-	-
4149	Hungary	cattle	I	4	8	-	-	-
4162	Hungary	sheep	I	2	16	-	-	-
4174	Hungary	cattle	I	1	2	-	-	-
4190	Hungary	cattle	I	2	4	-	-	-
4193	Hungary	cattle	I	1	1	-	-	-
4199	Hungary	cattle	I	1	1	-	-	-
4201	Hungary	cattle	S	4	4	-	-	-
4203	Hungary	cattle	I	1	2	-	-	-
4216	Hungary	cattle	I	4	2	-	-	-
4217	Hungary	cattle	I	8	4	-	-	-
4218	Hungary	cattle	R	32	16	-	-	-
4221	Hungary	cattle	R	64 <	64	-	+	+
4228	Hungary	rabbit	S	2	2	-	-	-
4231	Hungary	cattle	R	64 <	256 <	-	-	-
4246	Hungary	swine	S	0.25	0.25 >	-	-	-
4251	Hungary	cattle	R	64	64	-	-	-
4252	Hungary	sheep	I	1	8	-	-	-
4253	Hungary	cattle	I	2	4	-	-	-
4254	Hungary	cattle	I	1	1	-	-	-
4318	Hungary	goose	I	8	8	-	-	-
4319	Hungary	goose	I	4	8	-	-	-
4330	Hungary	goat	I	2	2	-	-	-
4340	Hungary	turkey	I	2	4	-	-	-

4341	Hungary	turkey	I	1	2	-	-	-
4373	France	duck	I	1	2	-	-	-
4376	France	duck	I	2	4	-	-	-
4380	France	duck	I	2	0.5	-	-	-
4385	France	duck	I	2	4	-	-	-
4389	France	turkey	R	2	4	-	-	-
4390	France	turkey	I	2	4	-	-	-
4400	France	turkey	R	0.5	2	-	-	-
4410	France	turkey	R	2	4	-	-	-
4473	Hungary	goose	I	2	4	-	-	-
4480	Hungary	goose	I	4	4	-	-	-
4483	Hungary	goose	I	4	4	-	-	-
4488	France	duck	I	2	2	-	-	-
4489	France	duck	I	1	2	-	-	-
4492	France	turkey	I	1	2	-	-	-
4499	France	albatross	I	0.5	1	-	-	-
4502	France	albatross	I	1	4	-	-	-
4539	Hungary	goose	I	16	0.5	-	-	-
4541	Hungary	goose	I	2	1	-	-	-
4542	Hungary	goose	S	2	0.5	-	-	-
4556	Hungary	goose	I	2	1	-	-	-
4564	Hungary	goose	I	2	2	-	-	-
4565	Hungary	goose	I	1	2	-	-	-
4573	Hungary	goose	I	1	2	-	-	-
4574	Hungary	goose	S	1	2	-	-	-
4576	Hungary	duck	R	2	4	-	-	-
4586	Hungary	fallow deer	S	1	2	-	-	-
4674	Hungary	turkey	I	2	4	-	-	-
4772	France	albatross	S	0.5	1	-	-	-
4924	France	turkey	I	4	32	-	-	-
4926	Hungary	turkey	I	1	4	-	-	-
5050	France	turkey	I	2	2	-	-	-
5144	Hungary	swine	I	0.5	0.5	-	-	-
	Resistance %		11.25	17.5	7.5	0.0	1.25	1.25

The last row shows the percentage of resistance detected in each antibiotic. Abbreviation: PEN: penicillin; AMX: amoxicillin; AMP: ampicillin; FUR: ceftiofur; CFX: cephalexin; GEN: gentamicin; TTC: tetracycline; DOX: doxycycline; ERY: erythromycin; CD: clindamycin; FFC: florfenicol; CHL: chloramphenicol; SMX: sulfamethoxazole; CPR: ciprofloxacin; ENO: enrofloxacin; UB: flumequine; TIL: tilmicosin; ARG: antibiotic-resistant gene; S: susceptible; I: intermediate; R: resistant; +: present; MIC: minimal inhibitory concentration; SNP: single-nucleotide polymorphisms; ARG: antimicrobial resistance gene; AA: amino acid.

Table S8. Phenotypic and genotypic antibiotic susceptibility profiles of P. multocida isolates across fluoroquinolones.

Strain ID	Country	Host species	Fluoroquinolones										
			Disk			MIC		AA substitutions in *par*C		AA substitutions in *gyr*A			
			CPR	ENO	UB	ENO (0.0015 - 16 µg/mL)	UB (0.06 - 64 µg/mL)	Glu84Lys	Ser80Leu	Ser83Ile	Ser83Arg	Asp87Asn	Asp87Gly
3029	Hungary	goose	S	S	I	0.06	64	-	-	-	-	-	-
3036	Hungary	goose	S	I	S	0.06	0.125	-	-	-	-	-	-
3083	Hungary	human	S	S	S	0.06	0.25	-	-	-	-	-	-
3171	Hungary	dog	S	S	S	0.06	0.25	-	-	-	-	-	-
3378	Hungary	cattle	S	S	S	0.015 >	0,06 >	-	-	-	-	-	-
3509	Hungary	cattle	S	S	S	0.03	0,06 >	-	-	-	-	-	-
3546	Hungary	sheep	S	S	S	0.015 >	0,06 >	-	-	-	-	-	-
3617	Hungary	goose	S	S	S	0.25	0.125	-	-	-	-	-	-
3687	Hungary	cattle	S	S	S	0.03	0.25	-	-	-	-	-	-
3699	Hungary	goat	S	S	S	0.015 >	0.125	-	-	-	-	-	-
3700	Hungary	goat	S	S	S	0.015 >	0,06 >	-	-	-	-	-	-
3770	Hungary	goose	S	R	S	0.06	0.125	-	-	-	-	-	-
3771	Hungary	duck	S	S	I	0.03	1	-	-	-	-	+	-
3903	Hungary	duck	S	S	S	0.03	0.5	-	-	-	-	+	-
4082	Hungary	goose	S	S	S	0.03	0.125	-	-	-	-	-	-
4107	Hungary	duck	S	S	S	0.06	1	-	-	-	+	-	-
4117	Hungary	swine	S	S	S	0.015 >	0.5	-	-	-	-	-	-
4122	Hungary	cattle	S	S	S	0.015 >	0,06 >	-	-	-	-	-	-
4125	Hungary	cattle	S	S	S	0.015 >	0,06 >	-	-	-	-	-	-
4138	Hungary	cattle	S	S	S	0.015 >	0.25	-	-	-	-	-	-
4144	Hungary	cattle	S	S	S	0.015 >	0,06 >	-	-	-	-	-	-
4145	Hungary	cattle	S	S	S	0.015 >	0,06 >	-	-	-	-	-	-
4147	Hungary	cattle	R	I	R	2	32	+	-	+	-	-	-
4148	Hungary	cattle	I	S	S	0.015 >	0,06 >	-	-	-	-	-	-
4149	Hungary	cattle	I	I	R	2	64	+	-	+	-	-	-
4162	Hungary	sheep	S	S	S	0.03	0.5	-	-	-	+	-	-
4174	Hungary	cattle	S	I	S	0.015	0,06 >	-	-	-	-	-	-
4190	Hungary	cattle	S	S	I	0.015 >	0.125	-	-	-	-	-	-
4193	Hungary	cattle	S	S	S	0.015 >	0,06 >	-	-	-	-	-	-
4199	Hungary	cattle	S	S	S	0.015 >	0.25	-	-	-	-	-	-
4201	Hungary	cattle	S	S	S	0.015 >	0,06 >	-	-	-	-	-	-
4203	Hungary	cattle	S	S	S	0.015 >	0,06 >	-	-	-	-	-	-
4216	Hungary	cattle	S	S	S	0.015 >	0,06 >	-	-	-	-	-	-
4217	Hungary	cattle	S	S	S	0.015 >	0,06 >	-	-	-	-	-	-
4218	Hungary	cattle	S	S	S	0.015 >	0.125	-	-	-	-	-	-
4221	Hungary	cattle	S	S	S	0.015 >	0.125	-	-	-	-	-	-
4228	Hungary	rabbit	S	S	S	0.25	1	-	-	-	-	-	+
4231	Hungary	cattle	I	I	R	2	16	+	-	+	-	-	-
4246	Hungary	swine	S	S	S	0.015 >	0,06 >	-	-	-	-	-	-
4251	Hungary	cattle	I	I	R	1	32	+	-	+	-	-	-
4252	Hungary	sheep	S	S	S	0.125	0,06 >	-	-	-	-	-	-
4253	Hungary	cattle	S	S	S	0.03	2	-	-	-	-	-	-
4254	Hungary	cattle	S	S	S	0.03	0.125	-	-	-	-	-	-
4318	Hungary	goose	S	S	S	0.015 >	0.25	-	-	-	-	-	-
4319	Hungary	goose	S	S	S	0.03	0.25	-	-	-	-	-	-
4330	Hungary	goat	S	S	S	0.03	0,06 >	-	-	-	-	-	-
4340	Hungary	turkey	S	S	S	0.015 >	0,06 >	-	-	-	-	-	-
4341	Hungary	turkey	S	S	S	0.015 >	0,06 >	-	-	-	-	-	-
4373	France	duck	R	R	S	0.015 >	0.125	-	-	-	-	-	-
4376	France	duck	R	S	R	0.015 >	0.125	-	-	-	-	-	-
4380	France	duck	S	S	I	0.06	1	-	-	-	-	+	-

4385	France	duck	S	S	I	0.125	4	-	-	-	-	+	-
4389	France	turkey	S	I	I	0.25	4	-	-	-	+	-	-
4390	France	turkey	I	I	I	0.015 >	0.125	-	-	-	-	-	-
4400	France	turkey	S	S	S	0.03	0.25	-	-	-	-	-	-
4410	France	turkey	I	I	I	0.015 >	0.125	-	-	-	-	-	-
4473	Hungary	goose	S	S	S	0.015 >	0.125	-	-	-	-	-	-
4480	Hungary	goose	I	S	R	0.5	64 <	-	+	-	+	-	-
4483	Hungary	goose	I	S	R	0.5	64	-	+	-	+	-	-
4488	France	duck	S	S	S	0.015 >	0.125	-	-	-	-	-	-
4489	France	duck	S	S	S	0.015 >	0.125	-	-	-	-	-	-
4492	France	turkey	S	S	S	0.015 >	0.125	-	-	-	-	-	-
4499	France	alba-tross	S	S	S	0.015 >	0,06 >	-	-	-	-	-	-
4502	France	alba-tross	S	S	S	0.015 >	0.125	-	-	-	-	-	-
4539	Hungary	goose	S	S	S	0.06	2	-	-	-	-	+	-
4541	Hungary	goose	S	S	R	0.125	4	-	-	+	-	-	-
4542	Hungary	goose	S	S	S	0.03	0.125	-	-	-	-	-	-
4556	Hungary	goose	I	I	S	2	4	-	-	+	-	-	-
4564	Hungary	goose	S	S	S	0.015 >	0.125	-	-	-	-	-	-
4565	Hungary	goose	S	S	S	0.015 >	0.125	-	-	-	-	-	-
4573	Hungary	goose	S	S	S	0.015 >	0,06 >	-	-	-	-	-	-
4574	Hungary	goose	S	S	S	0.015 >	0,06 >	-	-	-	-	-	-
4576	Hungary	duck	I	I	R	0.5	0.25	-	+	-	+	-	-
4586	Hungary	fallow deer	S	S	S	0.015>	0,06 >	-	-	-	-	-	-
4674	Hungary	turkey	S	S	S	0.015 >	0,06 >	-	-	-	-	-	-
4772	France	alba-tross	S	S	S	0.015 >	0,06 >	-	-	-	-	-	-
4924	France	turkey	S	S	I	0.06	1	-	-	-	+	-	-
4926	Hungary	turkey	S	S	I	0.015>	0,06 >	-	-	-	-	-	-
5050	France	turkey	S	S	S	0.03	0.125	-	-	-	-	-	-
5144	Hungary	swine	S	S	S	0.03	0.125	-	-	-	-	-	-
Resistance %			3.75	2.5	11.25	5.0	-	5.0	3.75	7.5	8.8	6.25	1.25

The last row shows the percentage of resistance detected in each antibiotic. Abbreviation: PEN: penicillin; AMX: amoxicillin; AMP: ampicillin; FUR: ceftiofur; CFX: cephalexin; GEN: gentamicin; TTC: tetracycline; DOX: doxycycline; ERY: erythromycin; CD: clindamycin; FFC: florfenicol; CHL: chloramphenicol; SMX: sulfamethoxazole; CPR: ciprofloxacin; ENO: enrofloxacin; UB: flumequine; TIL: tilmicosin; ARG: antibiotic-resistant gene; S: susceptible; I: intermediate; R: resistant; +: present; MIC: minimal inhibitory concentration; SNP: single-nucleotide polymorphisms; ARG: antimicrobial resistance gene; AA: amino acid.

Table S9. Phenotypic and genotypic antibiotic susceptibility profiles of P. multocida isolates across lincosamids.

Strain ID	Country	Host species	Licosamids		
			Disk	MIC	SNP
			CD	CD	23S rRNA
3029	Hungary	goose	R	32	+
3036	Hungary	goose	R	32	+
3083	Hungary	human	R	16	+
3171	Hungary	dog	R	16	+
3378	Hungary	cattle	R	32	+
3509	Hungary	cattle	R	128 <	-
3546	Hungary	sheep	R	32	+
3617	Hungary	goose	R	32	+
3687	Hungary	cattle	R	32	+
3699	Hungary	goat	R	128 <	+
3700	Hungary	goat	R	128 <	+

3770	Hungary	goose	R	32	+
3771	Hungary	duck	R	16	+
3903	Hungary	duck	R	32	+
4082	Hungary	goose	R	16	+
4107	Hungary	duck	R	16	+
4117	Hungary	swine	R	64	+
4122	Hungary	cattle	R	16	+
4125	Hungary	cattle	R	16	+
4138	Hungary	cattle	R	128 <	+
4144	Hungary	cattle	R	16	+
4145	Hungary	cattle	R	64	+
4147	Hungary	cattle	R	64	+
4148	Hungary	cattle	R	64	+
4149	Hungary	cattle	R	128 <	+
4162	Hungary	sheep	R	32	+
4174	Hungary	cattle	R	64	+
4190	Hungary	cattle	R	64	+
4193	Hungary	cattle	R	32	+
4199	Hungary	cattle	R	32	+
4201	Hungary	cattle	R	32	+
4203	Hungary	cattle	R	32	+
4216	Hungary	cattle	R	64	-
4217	Hungary	cattle	R	32	+
4218	Hungary	cattle	R	16	+
4221	Hungary	cattle	R	32	+
4228	Hungary	rabbit	R	32	+
4231	Hungary	cattle	R	128<	+
4246	Hungary	swine	R	32	+
4251	Hungary	cattle	R	128	+
4252	Hungary	sheep	R	32	+
4253	Hungary	cattle	R	32	+
4254	Hungary	cattle	R	16	+
4318	Hungary	goose	R	64	-
4319	Hungary	goose	R	32	+
4330	Hungary	goat	R	64	+
4340	Hungary	turkey	R	32	+
4341	Hungary	turkey	R	16	+
4373	France	duck	R	32	-
4376	France	duck	R	32	+
4380	France	duck	R	32	+
4385	France	duck	R	64	+
4389	France	turkey	R	64	+
4390	France	turkey	R	64	+
4400	France	turkey	R	32	+
4410	France	turkey	R	32	+
4473	Hungary	goose	R	32	+
4480	Hungary	goose	R	32	+
4483	Hungary	goose	R	32	+
4488	France	duck	R	32	+
4489	France	duck	R	32	+
4492	France	turkey	R	32	+
4499	France	albatross	R	32	+
4502	France	albatross	R	32	+
4539	Hungary	goose	R	32	+
4541	Hungary	goose	R	32	+
4542	Hungary	goose	R	32	+
4556	Hungary	goose	R	32	+
4564	Hungary	goose	R	16	+
4565	Hungary	goose	R	16	+
4573	Hungary	goose	R	32	+

4574	Hungary	goose	R	32	+
4576	Hungary	duck	R	32	+
4586	Hungary	fallow deer	R	32	+
4674	Hungary	turkey	R	32	+
4772	France	albatross	R	16	+
4924	France	turkey	R	16	+
4926	Hungary	turkey	R	32	+
5050	France	turkey	R	32	+
5144	Hungary	swine	R	16	+
	Resistance %		100.0	100.0	95.0

The last row shows the percentage of resistance detected in each antibiotic. Abbreviation: PEN: penicillin; AMX: amoxicillin; AMP: ampicillin; FUR: ceftiofur; CFX: cephalexin; GEN: gentamicin; TTC: tetracycline; DOX: doxycycline; ERY: erythromycin; CD: clindamycin; FFC: florfenicol; CHL: chloramphenicol; SMX: sulfamethoxazole; CPR: ciprofloxacin; ENO: enrofloxacin; UB: flumequine; TIL: tilmicosin; ARG: antibiotic-resistant gene; S: susceptible; I: intermediate; R: resistant; +: present; MIC: minimal inhibitory concentration; SNP: single-nucleotide polymorphisms; ARG: antimicrobial resistance gene; AA: amino acid.
